# From testbeds to high-stakes work: a review of Human-AI teaming domains and teaming factors

**DOI:** 10.3389/frobt.2026.1733942

**Published:** 2026-05-07

**Authors:** Shaida Kargarnovin, Christopher Ivan Hernandez, Dirk Reiners, Carolina Cruz-Neira, Grace Bochenek, Waldemar Karwowski

**Affiliations:** 1 Department of Industrial Engineering and Management Systems, University of Central Florida, Orlando, FL, United States; 2 Department of Computer Science, University of Central Florida, Orlando, FL, United States; 3 School of Modeling, Simulation, and Training, University of Central Florida, Orlando, FL, United States

**Keywords:** team processes, autonomy, communication and coordination, decision making, human-AI teaming, human-machine teaming, human-robot interaction, trust calibration

## Abstract

**Introduction:**

Human-AI teaming is increasingly being studied in applied and high-stakes settings, yet the evidence remains dispersed across domains, constructs, and research traditions. This fragmentation also limits efforts to connect broader human-AI findings to human-robot teaming (HRT), where embodied systems make issues such as coordination, autonomy management, communication, and safety more immediate in real-world interaction.

**Methods:**

To provide a clearer picture of the field, we conducted a PRISMA-guided systematic review with bibliometric analysis of 104 peer-reviewed empirical studies published between 2015 and 2025 and identified through Engineering Village, IEEE Xplore, PubMed, ScienceDirect, and Web of Science.

**Results:**

The review maps where human-AI teaming has been evaluated and what teaming aspects are most frequently examined. Cross-domain and interdisciplinary studies were the largest category, representing broad workplace or team-based investigations not tied to a single industry and instead focused on general collaboration issues such as communication, teamwork, coordination, and coworker interaction. Gaming and entertainment, aviation, military and defense operations, emergency response and public safety, and healthcare also represented substantial portions of the literature. Across studies, performance was the most frequently examined aspect, followed by trust, explainability and transparency, decision-making, and team processes. Bibliometric patterns suggest a shift since 2020 from foundational demonstrations in controlled settings toward applied, higher-stakes contexts where trust dynamics, communication, and ethical accountability more directly shape adoption and sustained performance.

**Discussion:**

Evidence points to a practical conclusion that human-AI teaming works best when the interaction supports coordination, allowing users to form accurate expectations of the AI, adjust autonomy and delegation across task phases, and use transparency cues that calibrate reliance without adding burden. For HRT, these findings reinforce the importance of shared control, mixed-initiative interaction, and designs that help humans and robots coordinate action over time rather than simply divide functions. We conclude by outlining implications for designing and evaluating human-AI teams as socio-technical systems and for prioritizing longitudinal and in-context studies that capture how teaming evolves over time.

## 1 Introduction

### 1.1 Background and scope of Human-AI teaming

Human-AI teaming involves collaborative work between humans and AI to achieve shared goals. This growing area of human-computer interaction (HCI) and computer-supported cooperative work examines how people perceive and trust AI teammates and how best to integrate AI to improve team performance (Zhang et al., 2021a).

This is especially transformative in embodied contexts, where AI takes the form of physical robots, i.e., human-robot teaming (HRT) and human-robot interaction (HRI). Robotic platforms introduce additional complexities such as spatial coordination, physical safety, and real-time adaptation in unstructured environments, making HRT a critical subdomain of human-AI teaming.

Effective human-AI teaming builds on complementary strengths between humans and AI. Humans excel in creativity, judgment, and social intelligence, while AI systems demonstrate exceptional capabilities in data processing, pattern recognition, and tireless performance. Together, these capabilities can significantly boost productivity and innovation (Bansal et al., 2019a; Kamar, 2016; Wang et al., 2016).

Key factors for effective human-AI teaming include shared mental models and a common understanding of tasks, goals, and expectations, which support coordination, action prediction, and smooth task flow (Ong et al., 2012; Zhang et al., 2021a). Human and AI teammates exchange information through natural language, visual cues, and shared data to exchange information, anticipate actions, and coordinate tasks (Lemon, 2022). Team-level processes such as implicit communication and joint decision-making further strengthen collaboration (Liang et al., 2019; Oh et al., 2018).

In addition to coordination, effective teaming depends on how humans perceive and evaluate their AI counterparts. Understanding and managing human expectations towards AI teammates is crucial because these expectations influence how responsibilities are allocated, how trust develops, and how the overall team dynamic unfolds (Ezer et al., 2019; Huang et al., 2021).

Human-AI teaming shows strong potential in many sectors. Examples include more accurate diagnoses and personalized treatment in healthcare (Watson et al., 2022), predictive maintenance and supply-chain optimization in manufacturing (Kohl et al., 2021), improved traffic routing and vehicle safety in transportation (Dhanare et al., 2022), and fraud detection plus tailored investment advice in finance (Alias et al., 2019).

Studies have shown that humans tend to exhibit biases towards their AI teammates, particularly when assigning blame for game failures (Merritt et al., 2011; Wingrove and Bond, 1998). Additionally, protection behaviors have been observed among human teammates in collaborative games involving AI partners (Ong et al., 2012). In military applications, people have struggled to work with AI due to cognitive limitations and shortcomings in technology design. It has been reported that there has been inadequate monitoring, situational awareness, trust calibration, and transparency. These findings underscore the importance of understanding and addressing human-AI biases and behaviors to optimize team performance and eliminate potential catastrophic failures. To enhance AI teaming collaboration, AI should no longer be viewed as a tool but as a genuine teammate (National Academies of Sciences et al., 2022).

As human-AI teaming continues to evolve, it holds the promise of revolutionizing various industries and enhancing human capabilities. By harnessing the complementary strengths of humans and AI, we can tackle complex challenges, optimize decision-making, and achieve unprecedented levels of productivity and innovation.

### 1.2 Related reviews and state of the art

Human-AI teaming has produced a growing number of literature reviews and syntheses in recent years, reflecting the field’s rapid development. The field of human-AI teaming has seen a surge in literature reviews and syntheses, particularly in recent years, reflecting its rapid maturation. Several systematic reviews have mapped key trends, frameworks, and applications up to 2025. For instance, a systematic literature review on human-AI collaboration in information systems research defines key features of collaborative decision-making and develops a conceptual three-dimensional framework for understanding human-AI interactions (Li et al., 2025). Similarly, a review and outlook on unraveling human-AI teaming builds on foundational views to reflect advanced AI capabilities, emphasizing AI agents’ ability to learn, adapt, and collaborate interdependently while requiring more cognitive input and coordination (Lou et al., 2025).

Domain-specific reviews have emerged as well. For example, in healthcare, one review examines human-AI teaming by distinguishing weak from strong complementarity. It notes that AI often augments clinician performance, with weak complementarity being common and strong complementarity being rare. Effectiveness depends on factors like teaming mode and clinician expertise (Liu et al., 2025). Another focused review explores key challenges such as lower trust, weaker shared cognition, and coordination issues in teams that include AI compared to human-only teams, and emphasizes the importance of factors like transparency, explainability, and reliable AI behavior for improving collaboration outcomes (Schmutz et al., 2024).

Broader reviews include work on trusting autonomous teammates in human-AI teams. For example, prior work has analyzed how trust is conceptualized and operationalized in existing human-AI teams studies and categorized factors influencing trust, particularly agent-related factors like transparency and reliability (Duan et al., 2025a). Additionally, a systematic literature review on artificial intelligence augmenting human teams identified opportunities for AI to enhance team coordination, while highlighting concerns such as over-reliance and ethical issues in collaborative settings (Khakurel and Blomqvist, 2022).

Recent literature has also considered different classes of AI systems in teaming contexts. For instance, a systematic review and taxonomy of human-agent teaming testbeds developed a taxonomy for human-AI teams by adapting existing frameworks originally designed for human teams. This taxonomy spans ten dimensions, including team composition and task environment (Chung et al., 2025). Another systematic review provides a taxonomy of interaction patterns in human-AI collaboration, proposing seven patterns that arise between humans and AI based on trends from reviewed studies (Gomez et al., 2025). Furthermore, a review on human control of AI systems analyzes supervisory human control and human-machine teaming as two main approaches, distinguishing levels of automation and autonomy. These reviews collectively represent the state of the art, shifting from early technical feasibility studies toward interdisciplinary explorations of trust, autonomy, and real-world integration across diverse AI classes (Tsamados et al., 2025).

#### 1.2.1 Human-robot teaming (HRT) reviews

Given that many high-stakes applications involve physically embodied AI, the literature also includes focused HRT syntheses. Foundational work by Wolf and Stock-Homburg synchronized definitions of HRT across psychology, engineering, and organizational perspectives, while clarifying the conditions under which robots can function as full team members (Wolf et al., 2020; Wolf and Stock-Homburg, 2023). Building on these foundations, Natarajan et al. articulated nine grand challenges for HRT, spanning fluent communication, human behavior modeling, long-term adaptation, scalability, safety, privacy, ethics, metrics, and human wellbeing (Natarajan et al., 2023). In military and first-response domains, Adams et al. mapped critical human roles to missions and identified key technical and organizational barriers to effective robotic teammates (Adams et al., 2024). More recently, Methnani et al. surveyed trustworthy AI in variable-autonomy robotic systems, stressing transparency and explainability for meaningful human control and trust calibration when autonomy levels shift dynamically (Methnani et al., 2024). Additional focused reviews address core technical enablers, including wearable-sensor-based task recognition for composite and concurrent tasks across multiple activity components (Baskaran and Adams, 2023) and mental modeling techniques that support mutual awareness and prediction in fluent HRT (Tabrez et al., 2020).

HRT reviews consistently suggest that one of the defining challenges of embodied collaboration is coordination under real-world constraints. Because humans and robots act in shared physical environments, successful teaming depends not only on communication but also on the ability to align actions in time, space, and purpose while adapting to changing task demands and safety requirements. This makes coordination in HRT more than a simple task division; it also involves anticipating a partner’s behavior, adjusting to their pace and intent, and maintaining a workable understanding of roles and responsibilities during joint activity. From this perspective, concepts such as mental models and theory-of-mind-related reasoning are often considered relevant because they help explain how teammates form expectations about one another’s actions, knowledge, and goals, which can reduce the need for constant explicit instruction and support more fluent collaboration (Hoffman et al., 2024; Methnani et al., 2024; Zhang and Williams, 2023).

### 1.3 Limitations of the existing reviews

Despite these contributions, existing reviews tend to be fragmented along disciplinary, conceptual, or application boundaries. Many focus narrowly on a single teaming factor (e.g., trust or explainability), a specific domain, or a particular class of AI systems. As a result, the broader structure of the human-AI teaming literature, how application domains relate to one another, which teaming factors dominate across contexts, and how research emphases have evolved over time, remain insufficiently synthesized. Furthermore, while some incorporate bibliometric tools or PRISMA guidelines, others lack rigorous temporal analysis or interdisciplinary integration, overlooking the socio-technical maturation from testbeds to real-world, high-stakes scenarios, particularly those involving embodied robotic platforms.

### 1.4 Objectives and contributions of this review

Despite the growing interest in human-AI teaming, there remains a lack of comprehensive understanding of its various application areas and the specific aspects that have been investigated. To address this gap, this literature review aims to provide a comprehensive overview of the research on human-AI teaming, guided by the steps discussed in the subsequent methodology section.

Complementing existing HRT-focused syntheses, we provide a broad, PRISMA-guided bibliometric review spanning both disembodied and embodied AI. The synthesis shows a clear shift since 2020 toward higher-stakes, applied settings, where trust, autonomy, communication, and ethics become central. By mapping where human-AI teaming has been evaluated, which teaming factors dominate across contexts, and how research emphases have evolved, this review provides actionable implications for designing and evaluating human-AI teams as socio-technical systems and for prioritizing longitudinal and in-context studies that capture how teaming evolves over time.

## 2 Methodology

This literature review adhered to the Preferred Reporting Items for Systematic Reviews and Meta-Analyses (PRISMA) (Moher et al., 2009).

### 2.1 Study design

We conducted a systematic review complemented by a bibliometric analysis to provide a comprehensive overview of human-AI teaming. A systematic review was selected for its rigorous, reproducible methodology, which includes quality assessment of studies, essential for synthesizing evidence in this emerging interdisciplinary field. Bibliometric analysis adds quantitative mapping of trends and networks, enabling a holistic view of the field’s evolution.

Our systematic literature review is driven by two key research questions (RQs):

RQ1: What are the key domains and contexts (e.g., healthcare, aviation, education) in which human-AI teaming has been applied and empirically evaluated in the literature?
RQ2: What core elements of human-AI teaming (e.g., trust, decision-making processes, performance outcomes, or ethical implications) have been examined in prior research?

Although a scoping review is suitable for exploratory mapping of broad topics, such as identifying application areas in human-AI teaming, we opted for a systematic review due to its integration with our other research question, which necessitates deeper evidence synthesis, including study quality evaluation and outcome appraisal. Scoping reviews typically emphasize breadth without formal quality assessment, whereas our systematic approach aligns with PRISMA guidelines (Moher et al., 2009), providing a robust foundation for bibliometric insights and actionable recommendations. This clarification highlights our rationale without dismissing the validity of a scoping approach (Arksey and O'Malley, 2005).

### 2.2 Search strategy

To capture the interdisciplinary nature of human-AI teaming, which spans health sciences and computer/engineering domains, we selected a diverse set of databases: Engineering Village and ScienceDirect for broad engineering and applied sciences coverage; IEEE Xplore for technical AI systems and interfaces; PubMed for biomedical and health-related literature (e.g., clinical human-AI applications); and Web of Science for citation-rich data supporting bibliometrics. This combination minimizes discipline-specific biases and maximizes recall, following best practices for systematic reviews that recommend using multiple, topic-relevant databases to capture diverse perspectives (Higgins et al., 2019).

Our search query focused on variations of “human-AI teaming” OR “human-AI collaboration” combined with “AND Teaming” where applicable. For transparency and reproducibility, the database-specific fields searched are as follows:

- Engineering Village: Searched in Title, Abstract, and Keywords.
- IEEE Xplore: Searched in Metadata (including title, abstract, and index terms/keywords).
- PubMed: Searched in Title/Abstract and MeSH terms (keywords).
- ScienceDirect: Searched in Title, Abstract, and Keywords.
- Web of Science: Searched in Topic (title, abstract, author keywords, and Keywords Plus).

In addition to database searches, backward citation searching was conducted by manually reviewing the reference lists of all full-text eligible articles. Searches were limited to studies published between January 2015 and August 2025, to focus on recent developments in this rapidly evolving field while capturing foundational empirical work.

### 2.3 Criteria for inclusion and exclusion

The subsequent inclusion criteria were employed: (a) English language papers; (b) peer-reviewed; (c) experiments in humans; (d) experiments investigating human-AI teaming.

**FIGURE 1:**
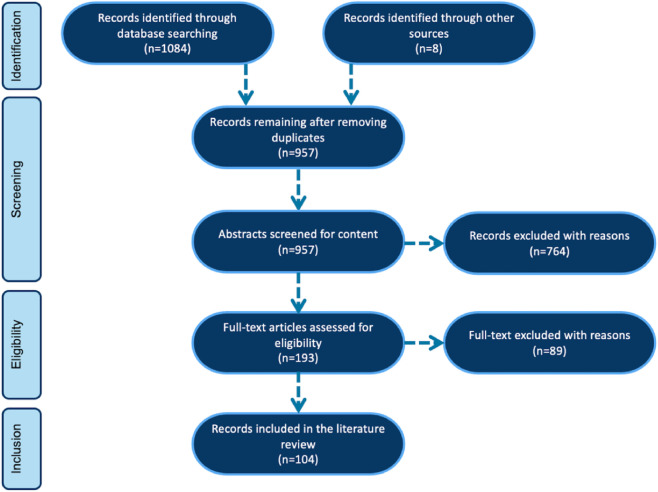
Flow diagram of the methods and selection processes used in this review, according to the PRISMA guidelines (Moher et al., 2009).

The subsequent exclusion criteria were employed: (a) opinions, viewpoints, and editorials without empirical data; (b) articles that did not pertain to the research questions; (c) papers that lacked original contributions, defined as those without novel empirical data, analyses, or insights (e.g., redundant summaries of existing work, literature reviews without new empirical findings or syntheses, non-peer-reviewed preprints, or purely descriptive overviews); (d) studies that did not include human-AI teaming; (e) studies that just proposed a framework without supporting experimental results or validation. The flow diagram of the methodology is shown in Figure 1.

A formal study-level quality appraisal was not conducted due to the heterogeneity of methodologies, domains, and study designs represented in the retrieved literature, which ranged from controlled laboratory experiments to field studies, simulations, and human-subject evaluations across multiple disciplines. Applying a single quality scoring instrument would have risked inappropriate or inconsistent assessments. Instead, rigor was ensured through strict inclusion criteria requiring peer-reviewed publication, empirical human-subject involvement, and direct relevance to human-AI teaming.

## 3 Results

### 3.1 Literature search

The literature review adhered to the PRISMA guidelines (Moher et al., 2009). As illustrated in Figure 1, a total of 1,084 records were initially identified through database searching, with 957 records remaining after duplicate removal. During the eligibility assessment, backward citation searching of reference lists from full-text articles identified eight additional relevant studies, which were assessed alongside the primary records. Following the application of the inclusion and exclusion criteria, a total of 104 studies were included in the final literature review. A summary list of the reviewed studies, organized by year, country, article type, investigated aspect of human-AI teaming, and application domain, is presented in Table 1.

**TABLE 1:** Summary list of the reviewed studies by year, country, article type, investigated aspect of Human-AI teaming, and application domain.

Study	Year	Country	Article type	Studied aspect	Application domain
Natarajan et al. (2024)	2024	USA	Conference paper	Autonomy	Emergency response
Paul et al. (2024)	2024	UK, Canada	Conference paper	Autonomy	Aviation, military and defense operations
Hauptman et al. (2025)	2025	USA	Journal article	Autonomy	Emergency response and public safety
Westphal et al. (2025)	2025	Spain	Journal article	Autonomy	Healthcare
Fuchs et al. (2024)	2024	Italy	Journal article	Autonomy	Transportation and driving
Veitch et al. (2022a)	2022	Norway	Journal article	Autonomy	Transportation and driving
Zhou and Gorman (2024)	2024	USA	Conference paper	Communication	Aviation
Xu et al. (2024)	2024	USA	Journal article	Communication	Engineering and design
Zahmat Doost et al. (2025)	2025	USA	Conference paper	Communication	Space operations
Zhang et al. (2024b)	2024	USA	Conference paper	Communication	Gaming and entertainment
Mallick et al. (2024)	2024	USA	Journal article	Communication	Gaming and entertainment
Gyory et al. (2021)	2021	USA	Conference paper	Communication, Problem-solving	Engineering and design
Bhatti et al. (2021)	2021	USA	Conference paper	Communication, Trust	Aviation
Zhang et al. (2023)	2023	USA	Conference paper	Communication, Trust	Gaming
Hu et al. (2025)	2025	USA	Journal article	Decision-making	Cross-domain and interdisciplinary
Chong et al. (2024)	2024	USA	Journal article	Decision-making	Engineering and design
Gou et al. (2024)	2024	China	Journal article	Decision-making	Supply chain management
Smith et al. (2025)	2025	Germany	Journal article	Decision-making	Security and identity
Fogliato et al. (2022)	2022	USA	Conference paper	Decision-making	Healthcare
Chong et al. (2023)	2023	USA	Journal article	Decision-making, AI Feedback, Confidence	Gaming
Tolmeijer et al. (2022)	2022	Switzerland	Conference paper	Decision-making, Ethics	Military and defense
Lauer and Wieland (2021)	2021	Germany	Conference paper	Decision-making, Forecasting	Supply chain management
Munyaka et al. (2023)	2023	USA	Conference paper	Decision-making, Team Efficacy	Gaming
De Brito Duarte (2023)	2023	Portugal	Journal article	Decision-making, Trust	Gaming
Textor et al. (2022)	2022	USA	Journal article	Decision-making, Trust, Ethics	Military
Gong and Zhang (2018)	2018	USA	Conference paper	Decision-making, Trust, Explainability	Cross-domain and interdisciplinary
Yu et al. (2017), Zhang et al. (2017)	2017	USA	Conference paper	Decision-making, Trust, and Explainability	Cross-domain and interdisciplinary
Zakershahrak et al. (2021)	2018	USA	Conference paper	Decision-making, Trust, Explainability	Emergency response
Dehkordi et al. (2021)	2021	UK	Conference paper	Decision-making, Trust, Explainability	Supply chain management
Jain et al. (2023)	2022	India	Journal article	Decision-making, Trust, Team Role Definition, Feedback Preferences	Cross-domain and interdisciplinary
Van den Bosch et al. (2025)	2025	Netherlands	Journal article	Ethics	Healthcare
Schelble et al. (2023b)	2023	USA	Conference paper	Ethics, Trust	Gaming
Schelble et al. (2022)	2022	USA	Journal article	Ethics, Trust	Military
Wang et al. (2024)	2024	China	Journal article	Explainability and Transparency	Cross-domain and interdisciplinary
Blaurock et al. (2024)	2024	Germany	Journal article	Explainability and Transparency	Cross-domain and interdisciplinary
Hauptman et al. (2024)	2024	USA	Journal article	Explainability and Transparency	Cross-domain and interdisciplinary
Darban (2024)	2024	USA	Journal article	Explainability and Transparency	Education
Momose et al. (2025)	2025	USA	Journal article	Explainability and Transparency	Emergency response and public safety
Paleja et al. (2024)	2024	USA	Conference paper	Explainability and Transparency	Gaming and entertainment
Bienefeld et al. (2024)	2024	Switzerland	Journal article	Explainability and Transparency	Healthcare
Zhang et al. (2024a)	2024	USA	Journal article	Explainability and Transparency	Military and defense operations
Paleja et al. (2021)	2021	USA	Conference paper	Explainability and Transparency	Gaming
Sadeghian and Hassenzahl (2022a)	2022	Germany	Conference paper	Job Satisfaction, Competence, Autonomy	Supply chain management
Li Y. et al. (2024)	2024	China	Journal article	Perception	Service and consumer
Flathmann et al. (2024a)	2024	USA	Journal article	Perception	Engineering and design
Flathmann et al. (2024b)	2024	USA	Conference paper	Perception	Gaming and entertainment
Attig et al. (2024)	2024	Germany	Conference paper	Perception	Gaming and entertainment
Schelble et al. (2025)	2025	USA	Journal article	Perception	Gaming and entertainment
Bendell et al. (2025)	2025	USA	Journal article	Perception	Military and defense operations
Kim J. et al. (2024)	2024	USA	Journal article	Perception, Team Processes	Education
Schecter et al. (2023)	2023	USA	Journal article	Perception, Transparency	Cross-domain and interdisciplinary
Figoli et al. (2022)	2022	Italy	Conference paper	Perception, Trust	Engineering and design
Aulia and Lin (2025)	2025	Taiwan	Journal article	Performance	Cross-domain and interdisciplinary
Lancaster et al. (2025)	2025	USA	Conference paper	Performance	Engineering and design
Flathmann et al. (2024a)	2024	USA	Conference paper	Performance	Gaming and entertainment
Chen et al. (2024)	2024	China	Journal article	Performance	Language
Islam et al. (2025)	2025	Canada	Journal article	Performance	Military and defense operations
Lu et al. (2023)	2023	USA	Conference paper	Performance	Education
Schoonderwoerd et al. (2022)	2022	Netherlands	Journal article	Performance	Emergency response and disaster response
Schwalb et al. (2022)	2022	USA	Conference paper	Performance	Emergency response and disaster response
Hauptman et al. (2023)	2023	USA	Journal article	Performance	Emergency response
Bansal, Nushi, Kamar, Weld, et al. (2019b)	2019	USA	Conference paper	Performance	Gaming
Kelly et al. (2023)	2023	USA	Conference paper	Performance	Gaming
Bansal et al. (2019a)	2019	USA	Conference paper	Performance	Healthcare
Bilgram and Laarmann (2023)	2023	Germany	Journal article	Performance	Supply chain management
Nguyen et al. (2021)	2021	USA	Conference paper	Performance	Service and consumer
Nguyen et al. (2022)	2022	USA	Conference paper	Performance	Service and consumer
Xu et al. (2023)	2023	USA	Conference paper	Performance	Cross-domain and interdisciplinary
Gopinath et al. (2022)	2022	USA	Conference paper	Performance	Driving
Nakahashi and Yamada (2021)	2021	Japan	Journal article	Performance, Autonomy	Gaming
Lemus et al. (2023)	2023	USA	Conference paper	Performance, Decision-making	Healthcare
Cao et al. (2023)	2023	USA	Conference paper	Performance, Decision-making	Cross-domain and interdisciplinary
Cabitza et al. (2021)	2021	Italy	Journal article	Performance, Decision-making	Healthcare
Ma et al. (2023)	2023	China	Conference paper	Performance, Decision-making, Trust	Cross-domain and interdisciplinary
Flathmann et al. (2023)	2023	USA	Journal article	Performance, Perception	Gaming
Zhao et al. (2022)	2022	USA	Journal article	Performance, Preference	Emergency response
Siu et al. (2021)	2021	USA	Conference paper	Performance, Preference	Gaming
Chong et al. (2022)	2022	USA	Conference paper	Performance, Self-confidence, Competence, Reliance on AI	Engineering and design
Zhang et al. (2022)	2022	USA	Conference paper	Performance, Strategy of Deception, Perceived Workload, Perceived Competency, Trust	Engineering and design
Hemmer et al. (2023)	2023	Germany	Conference paper	Performance, Task Satisfaction	Service and consumer
Schelble et al. (2023b)	2023	USA	Journal article	Performance, Team Cognition	Supply chain management
McNeese et al. (2021)	2021	USA	Journal article	Performance, Team Cognition, Situational Awareness	Emergency response
SharifHeravi et al. (2020)	2020	Australia	Conference paper	Performance, Trust	Aviation
Zhang et al. (2021b)	2021	USA	Conference paper	Performance, Trust	Gaming
Weitz et al. (2021)	2021	Germany	Conference paper	Performance, Trust	Gaming
Bienefeld et al. (2023)	2023	Switzerland	Journal article	Performance, Trust	Healthcare
Chen et al. (2022)	2022	China	Journal article	Team Cognition	Cross-domain and interdisciplinary
Farah and Dorneich (2024)	2024	USA	Conference paper	Team Processes	Emergency response and public safety
Kaelin et al. (2024)	2024	Sweden	Journal article	Team Processes	Healthcare
Edwards et al. (2024)	2024	USA	Journal article	Team Processes, Performance	Cross-domain and interdisciplinary
Harris-Watson et al. (2023)	2023	USA	Journal article	Team Processes, Receptivity of human teammates, Team Effectiveness	Cross-domain and interdisciplinary
Musick et al. (2021)	2021	USA	Journal article	Team processes, Team Cognition	Emergency response
Siemon (2022)	2022	Finland	Journal article	Team Role Definition	Cross-domain and interdisciplinary
Duan et al. (2024)	2024	USA	Conference paper	Trust	Aviation
Duan et al. (2025b)	2025	USA	Journal article	Trust	Aviation, military and defense operations
McWilliams et al. (2025)	2025	USA	Journal article	Trust	Cross-domain and interdisciplinary
Jorge et al. (2024)	2024	Netherlands	Journal article	Trust	Cross-domain and interdisciplinary
Love et al. (2024)	2024	Australia	Journal article	Trust	Cross-domain and interdisciplinary
Georganta and Ulfert (2024a)	2024	Netherlands	Journal article	Trust	Cross-domain and interdisciplinary
Georganta and Ulfert (2024b)	2024	Netherlands	Journal article	Trust	Cross-domain and interdisciplinary
Erengin et al. (2025)	2025	Netherlands	Journal article	Trust	Cross-domain and interdisciplinary
Li M. et al. (2024)	2024	USA	Journal article	Trust	Space operations
Hauptman et al. (2022)	2022	USA	Conference paper	Trust	Cross-domain and interdisciplinary
Li et al. (2023)	2023	USA	Journal article	Trust	Service and consumer

### 3.2 Overview of Human-AI teaming application areas and investigated aspects

To address the two research questions, we synthesized the included studies along two complementary dimensions: (i) the application contexts in which human-AI teaming has been empirically evaluated and (ii) the core teaming elements that these studies investigated. This section provides a descriptive overview of where human-AI teaming research has been conducted (RQ1) and what aspects of teaming have received the greatest empirical attention (RQ2). Detailed interpretations and implications of these patterns are further discussed in Section 4.

#### 3.2.1 RQ1: empirical application domains of Human-AI teaming

Table 2 summarizes the application domains represented in the reviewed studies. Domain classification was based on the application context explicitly described in each full-text article. Each study was assigned one primary domain reflecting its main task setting or intended use context. For example, drone, aviation, and defense studies were grouped under military and defense operations. Automated driving and other vehicle-related studies were coded as transportation. Triage and medical-advice settings were coded as healthcare, and product development and design tasks as engineering design. Thus, domains were not analytically merged but were standardized when different studies used related wording to describe the same broader application notion.

**TABLE 2:** Distribution of Human-AI teaming studies by application domain.

Application domain	Percentage
Cross-domain and interdisciplinary	22%
Gaming and entertainment	19%
Aviation, military and defense operations	12%
Emergency response and public safety	11%
Healthcare	9%
Engineering and design	8%
Supply chain management	6%
Service and consumer	5%
Education	3%
Transportation and driving	3%
Space operations	2%
Security and identity	1%
Language	1%

In contrast, studies classified as cross-domain and interdisciplinary did not specify a particular industry or applied context and were generally situated in broad workplace or team-based settings. They did not examine human-AI teaming within a specific niche industry or specialized application area. Instead, they addressed general aspects of collaboration that could be relevant across many domains.

The distribution of the studies suggests that cross-domain and interdisciplinary studies constitute 22% of the reviewed studies. Gaming and entertainment represent 19% of the studies and remain common experimental settings for investigating human-AI interaction under controlled conditions.

High-stakes application areas make up a substantial portion of the evidence base. Aviation, military, and defense operations account for 12% of studies, while emergency response and public safety account for 11%, and healthcare accounts for 9%. This distribution indicates that human-AI teaming research is increasingly being examined in operational settings where coordination quality can have direct safety and performance consequences.

Engineering and design account for 8% of studies, followed by supply chain management at 6% and service and consumer contexts at 5%, showing continued expansion into professional and organizational workflows. Smaller but established shares appear in education and transportation and driving, each at 3%, while space operations represent 2%. Security and identity and language-related applications each account for 1% and remain underrepresented, suggesting clear opportunities for future work in domains where AI becomes more deeply embedded in organizational and societal systems.

#### 3.2.2 RQ2: core elements investigated in Human-AI teaming

We used a short codebook to define the aspect categories used throughout the manuscript. These categories were developed as analytic lenses for synthesis, informed by the research questions and by recurring constructs in the included studies. The human-AI teaming coding framework used to classify the included studies is presented in Table 3.

**TABLE 3:** Human-AI teaming coding framework used to classify the included studies.

Manuscript category	Operational definition (what it covers)	Include if the study	Exclude if the study	Typical indicators for coding
Performance	Joint human-AI task outcomes, including effectiveness, efficiency, and robustness of team performance across changing conditions	Reports outcomes or perceived effectiveness, includes applied workflow impacts like productivity or quality control	Reports perceptions only, no outcomes	Accuracy, errors, time, productivity, completion rate, mission success, quality ratings, workload reduction, consistency across updates, resilience to failure, perceived effectiveness
Decision-making	How AI shapes judgments, choices, confidence, forecasting, decision quality	Measures decision quality, bias, confidence calibration, or how AI advice changes choices	Uses AI but does not measure decisions or advice use	Decision accuracy, choice shifts, confidence, calibration, forecasting accuracy, bias patterns, acceptance or rejection, advice taking, weighting, reliance
Team processes	How humans and AI coordinate as a team	Measures coordination, shared understanding, role clarity, interdependence, team dynamics, team competence	Single-user tool use with no teamwork constructs	Shared mental models, team cognition, role definition, coordination quality, team efficacy, collaboration behaviors
Trust	Trust in AI and related reliance, calibration, repair over time	Measures trust or uses reliance or compliance as a proxy, tracks trust dynamics	Uses trust as a general term without measures, focuses on explanations without trust or reliance outcomes	Trust scales, reliance, compliance, acceptance behavior, delegation willingness, calibration, repair after errors
Ethics	Fairness, accountability, responsibility, privacy, safety, harm in teaming	Evaluates ethical implications or perceptions, governance, responsibility in human-AI teaming	Mentions ethics only as background, technical fairness with no human or teaming component	Fairness or bias perceptions, accountability attribution, responsibility judgments, privacy concerns, safety or harm outcomes, ethical framing
Autonomy	Control allocation between human and AI, delegation, override, mixed initiative	Manipulates or analyzes autonomy level, handoffs, overrides, delegation decisions	Automation is present but autonomy or control allocation is not measured	Autonomy level, override frequency, delegation rate, handoff timing, authority perception, control satisfaction
Communication	How humans and AI exchange information for teamwork	Studies modality, strategy, feedback timing or content, turn-taking, interaction protocols	UI usability only, internal NLP metrics without human interaction evaluation	Grounding and repair, feedback usefulness, interaction fluency, turn-taking, message framing effects
Perception	Attitudes toward AI in teaming, acceptance, preference, satisfaction, perceived competence or agency	Measures acceptance, preference, satisfaction, perceived usefulness, competence, agency	Mainly trust or reliance outcomes where perception is not the focus, performance-only with no perception measures	Preference, satisfaction, acceptance or intention to use, perceived usefulness, perceived competence, perceived agency, attitude scales
Explainability an transparency	User-facing information about AI reasoning or state	Tests explanation forms, uncertainty displays, rationales, interpretability features with user outcomes	Claims interpretable but not user-facing, technical interpretability without user or team evaluation	Explanation satisfaction, understanding, mental model accuracy, uncertainty comprehension, rationale usefulness, transparency effects on teaming outcomes

To support consistency, a subset of studies sampled across domains and publication years was independently coded by a second reviewer. Disagreements were resolved through discussion and reference to the codebook definitions, and recurring ambiguities were used to refine the codebook before coding the remaining studies. The codebook should therefore be understood as a structured interpretive tool for organizing a heterogeneous literature, rather than as a definitive or mutually exclusive taxonomy of human-AI teaming.

The human-AI teaming literature is conceptually interdependent, and overlap exists among these related constructs. To improve consistency in classification, we conducted an initial pilot pass in which category definitions were refined to resolve borderline cases. Each study was then assigned one primary aspect, the central construct driving its aims, hypotheses, and primary outcome measures, while any additional measured or manipulated constructs were recorded as secondary aspects. When authors used different terms for closely related ideas, such as reliance and trust, studies were classified according to their operationalization rather than surface terminology.

**FIGURE 2:**
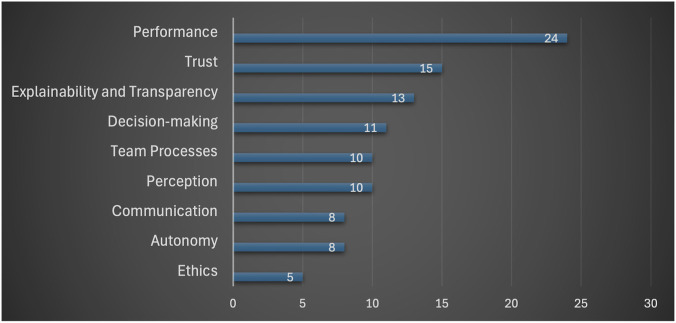
Frequency chart of application areas/domains in the included studies.

Figure 2 provides a frequency-based summary of the core aspects examined: performance, trust, explainability and transparency, decision-making, team processes, perception, communication, autonomy, and ethics.

Performance emerged as the most frequently examined aspect (24 studies), followed by trust (15), explainability and transparency (13), decision-making (11), team processes (10), and perception (10). Communication (8), autonomy (8), and ethics (5) were also represented. Overall, the distribution suggests that the literature remains anchored in performance and trust while also expanding toward coordination, interpretation, control, and broader socio-technical conditions of human-AI collaboration.

### 3.3 Large-scale bibliometric analysis

VOSviewer software was employed for our bibliometric analysis (Van Eck and Waltman, 2010). VOSviewer’s bibliometric network mapping capabilities enable the visualization and analysis of publication trends in specific fields. Using VOSviewer, one can create networks of scientific publications, journals, co-authorship, countries, and keyword co-occurrences.

**FIGURE 3:**
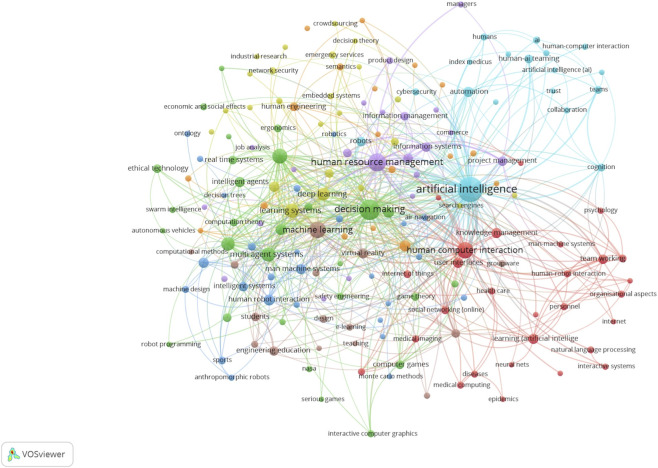
Network visualization of keyword co-occurrence.

**FIGURE 4:**
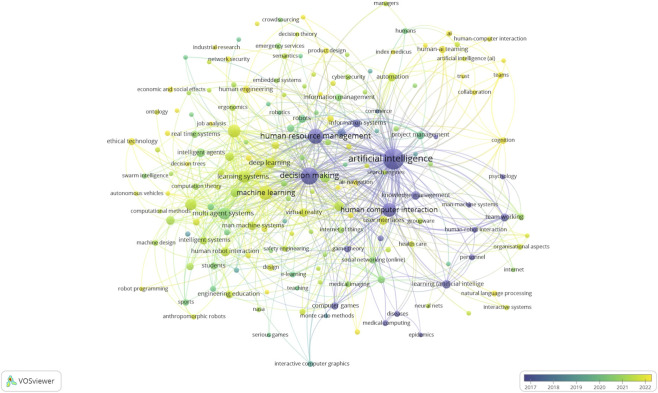
Overlay graph of the temporal evolution of human-AI research.

To provide a comprehensive bibliometric perspective, two complementary network visualizations were generated using VOSviewer. The first network (Figure 3) represents a keyword co-occurrence map that captures the structural relationships among major research themes in human-AI teaming. The second network (Figure 4) is an overlay visualization that builds on the same co-occurrence structure but incorporates temporal information, allowing examination of how thematic emphases have evolved over time.

To gather the data for the analysis, we downloaded each database result in the. ris format and merged them into a single file using Zotero citation management software. After removing duplicates and refining the citations, we exported the. ris file from Zotero and used it as the data file for VOSviewer. In the co-occurrence analysis of keywords, we set the minimum number of occurrences for a keyword to 10. Based on the selected criteria, VOSviewer generated a network visualization graph depicting the analysis of keywords (Figure 3).

Objects are marked and encircled based on their weight. Objects with greater weights are assigned larger labels and circles. Connections in the VOSviewer network signify the simultaneous occurrence of two keywords, and the proximity of keywords indicates a more robust relationship. The numerical value assigned to each link in the VOSviewer network signifies the intensity of the association, with stronger links having higher values. This value is determined by tallying the instances in which two keywords co-occur in publications.

In the network visualization map shown in Figure 3, each node is a keyword from the retrieved records. Node size reflects how often that keyword appears in the records. Links connect pairs of keywords that co-appear in the same records; thicker links indicate stronger co-occurrence. Shorter distances between nodes approximate higher relatedness. Colors denote clusters, which are groups of keywords that tend to co-occur more with each other than with the rest of the network. Together, they reveal the main topical sub-areas represented in the search results.

The bibliometric network presented in Figure 3 reveals four major clusters that outline the structure of human-AI teaming research.

The blue cluster, centered on artificial intelligence, links strongly to human-AI teaming, trust, collaboration, cognition, and automation. This cluster captures the conceptual core of the field, showing how AI is studied as both a technical enabler and a collaborative partner. The presence of project management and efficiency indicates translation into applied settings where coordination and performance are critical, tying directly to our categories of Performance, Decision-making, and Trust.

The red cluster, anchored in HCI, highlights the role of user interfaces, HRT, team working, healthcare, and organizational aspects. These terms emphasize the importance of interaction design, communication strategies, and team processes in domains where safety and clarity are vital. The inclusion of healthcare underscores the relevance of human-AI teams in high-stakes environments, while team working and organizational aspects connect this body of work to broader questions of workforce integration. This cluster aligns with our themes of Communication, Perception, and Ethics.

The green cluster, structured around decision-making and machine learning, extends to multi-agent systems, intelligent agents, behavioral research, and autonomous agents. Dense co-occurrence here reflects the technical backbone of human-AI teams, where algorithmic advances support autonomy, delegation, and coordination. The proximity of HRT, engineering education, and students suggests that much of this work is tied to robotics, training, and adaptation, bridging technical development with human learning.

The purple cluster, led by human resource management and information systems, represents the organizational and managerial lens of human-AI teaming research. Connections to cybersecurity, ergonomics, product design, and employment reflect concerns with system resilience, workforce adaptation, and human-centered design. These studies illustrate how AI adoption affects not only performance but also the structure of work and management practices, resonating with our categories of Team Processes and Perception.

Overall, these clusters demonstrate that research on human-AI teaming is multifaceted and interconnected. Technical advances in learning systems and autonomy (green) feed into questions of performance and trust (blue), which rely on effective interfaces and communication strategies (red), all situated within organizational and workforce contexts (purple). This structure mirrors the categories in our review and shows how the literature collectively approaches human-AI teaming from complementary perspectives.

To examine the evolution of these themes over time, we generated an overlay visualization (Figure 4) of the same co-occurrence network shown in Figure 3. In this overlay, node colors represent the average publication year of documents containing each keyword (darker shades for earlier years, brighter for recent ones).

The overlay visualization highlights the temporal evolution of research within human-AI teaming. Earlier work (darker purple/blue nodes, 2017-2019) concentrated on foundational themes such as artificial intelligence, HCI, decision-making, and machine learning, establishing the technical and conceptual backbone of the field.

More recent studies (green to yellow nodes, 2020 onward) extend toward applied and emergent areas. Keywords such as human-AI teaming, trust, collaboration, and healthcare appear in brighter shades, indicating their growing prominence in the past 3 years. Similarly, autonomous agents, ethical technology, and network security mark expanding attention to autonomy, ethics, and resilience.

This temporal shift shows a progression from broad system-level questions toward more domain-specific and socially embedded concerns. Where early research built the methodological and technical foundation, recent work emphasizes integration into high-stakes contexts and explores the human and organizational dimensions of AI collaboration.

## 4 Discussion

The discussion is organized by the main teaming aspects identified in the reviewed studies. Across these sections, we emphasize where evidence converges, where it diverges, and which task and design conditions explain those differences. We then synthesize these findings to outline their broader implications for human-robot teams, where embodiment introduces additional coordination, safety, and control challenges.

### 4.1 Performance

In this review, performance refers to how well the human-AI team functions as a joint system, not simply to the standalone accuracy of either the human or the AI. More specifically, performance includes three related dimensions: effectiveness, or whether the team achieves accurate, high-quality, or otherwise successful outcomes; efficiency, or whether those outcomes are achieved with less time, effort, or friction; and robustness of performance, or whether the team can maintain effective functioning across changing conditions, repeated interaction, system updates, or uncertainty. This distinction is important because many studies in the literature do not simply ask whether the AI is accurate, but whether the interaction allows human and AI strengths to combine in ways that improve task success, reduce coordination costs, and sustain effective collaboration over time. In the discussion below, factors such as mental models, transparency, training, and delegation are therefore treated not as performance itself, but as mechanisms that shape these performance outcomes.

Several studies point to mental models and predictability as the mechanisms behind performance. Using the CAJA platform, Bansal et al. show that updates intended to improve AI accuracy can still reduce team performance if they disrupt the user’s learned model of the AI’s behavior; the highest team performance emerges when people develop stronger mental models, and the system supports compatibility across updates (Bansal et al., 2019a). Related CAJA findings further suggest that it is not only how accurate the AI is, but how its errors are structured, with more stable and parsimonious error boundaries making it easier for people to anticipate failures and collaborate effectively, and those mental models take repeated interaction to develop (Bansal et al., 2019b). Together, these results imply that when AI behavior changes (through updates or shifting distributions), human-AI performance can drop even if model accuracy improves, unless the update preserves or repairs users’ predictive understanding of the system.

This same logic shows up outside CAJA. In a gaming setting, Siu et al. report that users preferred the partner that was easier to coordinate with (SmartBot), even though team performance did not meaningfully differ, again emphasizing that predictability and coordination can matter as much as raw capability in determining how well a human-AI team functions (Siu et al., 2021). Zhao et al. reinforce this point in a navigation/search-and-rescue setting. Adaptivity does not raise performance overall, but when instruction complexity matches user knowledge, it reduces confusion and frustration and improves engagement and confidence, suggesting that “better” AI is often “better fit” not necessarily “more complex” (Zhao et al., 2022). A recurring implication across performance studies is that “better AI” is not a sufficient condition for better teaming. Performance often improves when the interaction protocol is designed to convert individual judgments into reliable team decisions. In other words, the protocol can turn relatively weaker individual decision-makers into a stronger team by structuring aggregation and review in a way that leverages complementarity and reduces individual error. This supports the broader claim that performance in human-AI teams is often a property of the interaction protocol, how judgements are combined, when AI is consulted, and how disagreement is resolved, rather than a simple reflection of the AI model’s standalone accuracy (Cabitza et al., 2021).

Transparency is not a guaranteed performance boost. Across the reviewed studies, explanation and confidence cues help when users can interpret them and when the system’s behavior is stable enough to learn; they hurt when cues increase cognitive load, amplify over-reliance, or are offered to users who lack the expertise to act on them effectively (Nguyen et al., 2021; Nguyen et al., 2022; Tejeda Lemus et al., 2023). Under time pressure, this risk is sharper because people may lean on AI suggestions even while judging the AI as less useful, which can degrade decision quality and downstream performance (Cao et al., 2023). Overall, transparency works best when it is calibration-focused (helping people know when to trust) rather than information-heavy.

Performance evidence from embodied and high-stakes contexts suggests that gains are driven less by “better predictions” and more by (i) alignment of intent and situation understanding, (ii) dynamic delegation, and (iii) autonomy that matches task phase and consequences. First, when tasks are spatial, time-sensitive, and partially observable (e.g., search-and-rescue), performance improvements track whether the interaction helps teams build a shared account of what the agent is doing and why. Protocols that force brief explanation, confirmation, or structured dialogue appear to improve coordination and memory for agent actions, which is a prerequisite for effective delegation and error recovery in these environments (Schoonderwoerd et al., 2022; Schwalb et al., 2022).

Second, embodied teaming performance is strongly shaped by delegation behavior over time. People do not “use autonomy” once; they negotiate it continuously. Across simulated rescue settings, participants tend to increase autonomous-mode use as familiarity grows, and incentives can shift how much responsibility humans are willing to hand over; this means that performance outcomes reflect not only system capability, but adoption dynamics and the conditions under which humans choose to delegate (Schoonderwoerd et al., 2022; Schwalb et al., 2022). In practical terms, this makes delegation a performance variable. The same AI can look effective or ineffective depending on whether the work design and feedback loops support sustained, appropriate use.

Third, high-stakes teaming repeatedly points to autonomy-phase fit, where higher autonomy is more acceptable and less disruptive in routine, predictable phases, while lower autonomy is preferred when the work becomes uncertain, irreversible, or requires nuanced trade-offs. This phase-sensitive pattern shows up clearly in cyber incident response (where later actions have higher consequences and ambiguity) and is consistent with the broader idea that autonomy should be dialed to the stability of the process and the cost of errors (Hauptman et al., 2023a). Related work in driving and other dynamic contexts reinforces the same point from a design angle, showing that teams benefit when the system is built to model human behaviors and tailor support policies, even if the contribution is mainly methodological/simulation-based rather than a direct field performance benchmark (Gopinath et al., 2022).

Finally, several studies in applied settings are best interpreted as workflow augmentation rather than direct performance evaluation, because they clarify where AI fits into real work (e.g., innovation, content production, instructional design), and they show that “performance” often includes time, iteration cost, and quality control requirements rather than a single accuracy metric (Bilgram and Laarmann, 2023; Lu et al., 2023). At the same time, applied task studies show why “fit” and “incentives” belong inside the performance claim. Xu et al. demonstrate that AI can make work objectively easier and faster (e.g., improved completion time/recall via recommendation behavior), yet users may still prefer manual methods, highlighting that incentives, perceived control, and the cost of verification are part of performance in deployed human-AI teams (Xu et al., 2023).

These applied findings set up a broader point, that performance is shaped by the human role and by the conditions that make teaming sustainable, not only by the immediate task setting. Hemmer et al. show that AI delegation can improve both performance and satisfaction, with self-efficacy mediating the effect, suggesting that performance gains can be achieved when the system increases users’ sense of capability and control rather than replacing them (Hemmer et al., 2023). In contrast, Schelble et al. report cases where adding an AI teammate reduced performance relative to human-only teams, indicating that teaming can fail when coordination costs exceed benefits, especially under higher task difficulty or when the AI teammate is perceived as artificial or hard to align with (Flathmann et al., 2023).

Evidence also suggests that mental-model problems persist even when people receive repeated exposure and feedback. Kelly et al. show that individuals tend to overestimate AI teammates’ performance, expect AI abilities to be strongly correlated across problem types, and fail to fully learn the agent’s specific strengths and weaknesses over time. This matters for performance because inaccurate mental models can push teams toward the wrong coordination strategy, either over-delegating when the AI is weak in a subtask or underusing it when it is strong, reducing complementarity even when the AI is capable (Kelly et al., 2023).

In organizational settings, the same sustainability issue appears as a socio-technical one. Evidence from remote work suggests that stronger AI-assistant skills are associated with better human-AI teaming quality and task-technology fit, which supports wellbeing and work engagement over time. While engagement is not the same as task accuracy, it is a meaningful performance pathway in real work. It captures whether AI use is sustainable, whether it reduces friction rather than adding it, and whether teams can maintain effective collaboration (Aulia and Lin, 2025). This connects directly to the training evidence in the performance literature. Lancaster et al. (2025) report that teammate-model training produces better long-term performance and higher satisfaction than training that frames AI as merely a tool, implying that people benefit from explicit coordination strategies and expectations-setting when collaborating with AI (Lancaster et al., 2025). Flathmann et al. add nuance by suggesting that purely collaborative (human-human) training may not transfer well to later human-AI teaming because it can lock in shared mental models that do not match AI partners, reducing later human-AI teams’ performance (Flathmann et al., 2023). Complementary evidence on adaptation suggests that not all adaptation improves performance. Chen et al. indicate that affective adaptation (liking the AI) can reduce emotional adjustment in ways that hurt individual performance, while perceptions that the AI is intelligent, usable, and explainable support cognitive/behavioral adaptation that improves collaboration and performance (Chen et al., 2024). More generally, performance in complex human-AI teams appears to peak when humans provide targeted corrections while the AI retains control of execution. Islam et al. illustrate this with policy correction, which improves learning outcomes while reducing human workload compared with full manual control, supporting the idea that “middle-ground” oversight can outperform both human-only and AI-only baselines (Islam et al., 2025).

Taken together, the applied literature supports a narrower, more defensible claim that in high-stakes teaming, performance improves when interaction design makes the AI predictable to coordinate with, delegation is supported and revisable, and autonomy is matched to task phase and consequences, not simply when the underlying model is more accurate.

### 4.2 Decision-making

AI is increasingly embedded in decision workflows, but the reviewed studies suggest that decision quality depends less on “adding AI” and more on how the collaboration is configured. Across domains, decision-making outcomes consistently hinge on (i) whether the task is structured or judgment-heavy, (ii) how AI output is integrated into the workflow, (iii) how confidence and feedback are presented, and (iv) whether the team can maintain calibrated trust over time.

A first recurring pattern is that users respond differently to AI depending on task structure. For structured, routine work, automation tends to be seen as appropriate and desirable; for unstructured, high-judgment decisions, the same automation can be perceived as a black box and resisted. In those cases, “augmentation” is often preferred because it allows tailoring, although tailoring introduces learning costs and new opportunities for user error. This points to a practical design implication, namely that automation fits structured tasks, while augmentation fits judgment-heavy tasks, and blended approaches may be necessary across a single workflow (Gou et al., 2024). Consistent with this, performance improves when AI integration reduces friction in the decision pathway rather than adding a separate AI step. In clinical decision contexts, aligning AI inferences with human reasoning through a workflow that supports quick reconciliation can improve diagnostic outcomes, reinforcing the broader idea that workflow design is a core determinant of decision performance (Fogliato et al., 2022).

A second pattern is that decision-makers anchor on AI advice in ways that are sensitive to perceived AI quality, feedback, and cognitive load. Confidence and explanation cues can increase reliance, but they do not reliably improve outcomes when they overload users or encourage reliance under weak AI conditions (Chong et al., 2023). The risk becomes sharper when decision-makers face multiple AI teammates. Evidence suggests that confidence can decline over time even with high-performing AI, and that feedback about one AI can inadvertently affect perceptions of another, creating cross-effects that look like cognitive overload rather than rational calibration. This cautions against assuming that adding more AI advisors automatically improves trust or acceptance (Chong et al., 2024).

Several studies also highlight that agreement structure is a dominant cue in human-AI decision behavior. When AI and a human teammate agree, people are much more likely to accept the recommendation; when they disagree, people often defer to the human. Allowing users to choose whether to view AI advice can increase uptake among those who consult it, but has little effect if users routinely skip it, implying that optional advice is only effective when team structures encourage seeking it out at relevant times (Smith et al., 2025).

Voting structures add another layer. Being outvoted by AI agents can shift confidence and emotions depending on outcomes, and performance depends sharply on the quality of the AI advisors. Strong agents can help, but even one weak agent can harm outcomes. Over time, people may also re-weight their own judgment and rapidly discount low-performing advisors, suggesting that multi-advisor designs must actively manage quality differences and the psychological consequences of being outvoted (Hu et al., 2025).

Across the decision-making literature, trust appears less like a static attitude and more like an outcome of what users can interpret and how the work is organized. Transparency can increase trust (especially in higher-risk choices) when it helps users make sense of the AI’s recommendation and when it supports acceptance without forcing excessive cognitive effort (De Brito Duarte, 2023). At the same time, trust is shaped by work design, role clarity, collaboration pattern (e.g., side-by-side workflows), and the feedback users expect all influence whether trust stabilizes or erodes across repeated interactions (Jain et al., 2023).

A consistent implication is that trust is not something to maximize; trust is improved when it becomes calibrated rather than simply higher. Confidence-aware workflows that adapt who contributes what based on moment-to-moment uncertainty can strengthen both team performance and appropriate trust by preventing over-reliance when the system is unsure and preserving human agency when judgment is needed (Ma et al., 2023).

Finally, decision-making in real work often depends on how knowledge is distributed within the team. In forecasting and planning contexts, collaboration benefits from complementarity (humans contribute contextual understanding while AI contributes data-driven patterns) but information asymmetry can limit this benefit when humans lack access to the same information as the AI or when the AI lacks local context that humans have. This implies that effective collaboration requires careful design of how knowledge is surfaced within the team (Lauer and Wieland, 2021).

To improve knowledge distribution, it's important to consider interpersonal and team dynamics. Disclosing partner identities and decision-making styles can influence both individual and team confidence. This highlights that, even in hybrid teams, building rapport and establishing clear communication expectations are crucial for shaping how people interact with AI support over time (Munyaka et al., 2023).

The main implication points to a clear design direction for calibration under disagreement, managing cognitive load in multi-advisor settings, and structuring feedback to help users can learn which source is reliable for which kind of decision.

### 4.3 Team processes

Across the reviewed studies, team processes are less about the AI’s raw capability and more about whether the team develops the coordination conditions that make collaboration work. The literature repeatedly points to three practical levers. Teams benefit when coordination and attention are well distributed, when the AI is perceived as a viable teammate, and when roles and interaction norms are clear enough to prevent avoidable friction over time.

#### 4.3.1 Team dynamics and competence

A consistent pattern is that collaboration quality depends on how humans calibrate their reliance on AI and how they interpret the AI’s competence relative to their own. In engineering design contexts, self-confidence is tied to reliance behavior, and reliance can be beneficial when it reflects appropriate use of AI input rather than disengagement or blind acceptance (Chong et al., 2022). At the same time, attempts to manage collaboration through deception cues do not reliably improve joint work and can harm collaboration for less proficient users, suggesting that team dynamics are sensitive to perceived legitimacy and fairness (Zhang et al., 2022). These findings point to an important boundary. Competence signals matter, but calibration should be achieved through transparent, user-respecting design rather than manipulation.

Team dynamics also show up in information-heavy work, where AI can improve team processes by shifting effort and attention. AI can reduce effort and speed identification by prioritizing which items receive attention, reinforcing the idea that process improvements often come from better division of labor and reduced coordination burden (Edwards et al., 2024).

#### 4.3.2 Team cognition

Team cognition results converge on a straightforward mechanism. Human-AI teams function better when the interaction strengthens shared situational awareness and when coordination strategies make use of human-AI differences rather than trying to reproduce human-only teamwork. In emergency-response simulations, team performance and situational awareness are closely linked, and the presence of an AI teammate changes how humans coordinate and synchronize their actions (McNeese et al., 2021). This helps explain why adding an AI teammate does not automatically improve outcomes, since team cognition emerges from interaction patterns rather than capability alone.

Several studies suggest that AI can strengthen team cognition when it supports teamwork behaviors directly rather than only producing task outputs. When AI advice is well-timed and oriented toward team processes such as resource allocation and maintaining shared awareness, teams can report higher satisfaction and improved performance, consistent with the idea that AI can act as a teamwork coach that scaffolds shared mental models (Farah and Dorneich, 2024).

Musick et al. (2021) explored how perceived team composition affects sentiments, team processes, cognitive states, and the emergence of team cognition in human-autonomy teaming. Their findings revealed thematic differences between teams with only human members and those believing their teammates were autonomous agents. Interestingly, participants in teams with AI teammates reported more positive perceptions of their teammates and demonstrated more effective coordination and communication. These results suggest that human perceptions of AI teammates can influence human-agent teaming performance and that interventions like enhanced experience, education on AI capabilities, and training can help address social cognition and biases against teaming with autonomous agents (Musick et al., 2021).

Team cognition is especially relevant to understanding coordination in human-AI and human-robot teams because effective teamwork depends not only on what each teammate does individually, but also on how teammates represent the task, interpret one another’s roles, and anticipate likely actions during joint activity. In this sense, coordination is more than general collaboration or communication; it involves the ongoing alignment of interdependent actions in response to changing task conditions. Shared mental models are central to this process because they support common expectations regarding goals, responsibilities, procedures, and task progress, enabling teammates to adapt with less reliance on constant explicit instruction. Theory-of-mind-related reasoning is likewise relevant, particularly in embodied teaming contexts, because it refers to the capacity to infer another agent’s beliefs, intentions, knowledge, or likely next actions and to use those inferences to guide one’s own behavior. In teamwork, this kind of reasoning supports anticipation, action prediction, timing, and the selection of responses that are sensitive to a partner’s likely needs or limitations. In human-robot teams, theory-of-mind-related capabilities are especially important because fluent collaboration often depends on whether the robot can model human intent, recognize when a person may need assistance, predict how a teammate is likely to act, or adjust its behavior in ways that remain understandable and useful to the human partner. Together, shared mental models and theory-of-mind-related reasoning help explain how teams move beyond simple information exchange toward more anticipatory and coordinated interaction (Butchibabu et al., 2016; Eduardo et al., 2005; Hoffman et al., 2024; Salas et al., 2008; Schröder et al., 2025).

#### 4.3.3 Team role definition

The effective integration of AI into teams depends on the precise definition of AI team member roles and responsibilities. Individuals often begin by conceptualizing AI as a tool rather than a teammate, but their role expectations can expand when prompted, indicating that role concepts are flexible and can be shaped by design and experience (Siemon, 2022). Role ambiguity increases coordination costs. When people are unsure if an AI is advising, executing, or sharing responsibility, teams spend time resolving friction instead of focusing on the task. Further, even if an AI is capable, teams may not progress into stable cooperative norms without explicit support for conflict management. In human-AI teams, participants often experience early-stage conflict, and some fail to move into a working relationship, reporting that they remained stuck. Those who do stabilize emphasize user leadership and personalization as conditions for effective teamwork (Kaelin et al., 2024). This aligns with classic group-development accounts of teams passing through conflict before stabilizing cooperation (Tuckman, 1965), but it also suggests that human-AI teams might require more structured support to facilitate their collaboration effectively.

Finally, social perception studies show why role definition and cognition cannot be separated from human beliefs about the teammate. When people perceive AI teammates as competent and warm, they are more receptive and view them as viable collaborators, with competence typically doing more of the work than warmth (Harris-Watson et al., 2023). Yet there is a counterintuitive finding that keeps this from becoming a simple “make the AI likable” story. Emotional preference for AI as a colleague can be associated with greater conflict behavior, while perceived intellectual ability is associated with reduced conflict, suggesting that affective attachment and perceived competence may push team behavior in different directions (Chen et al., 2022). The implication is that teams benefit from cues that support credible competence and predictability, not just social appeal.

This sensitivity to perception is reinforced by evidence that team processes shift based on what people believe the team composition is. When participants think autonomous agents are part of the team, sentiments and coordination patterns can change, sometimes in positive directions, indicating that expectations and social framing alter how teams communicate and organize around autonomy (Musick et al., 2021). Taken together, these studies suggest that team processes in human-AI teams are shaped by both coordination design and social cognition. The same technical capability can support collaboration or strain it, depending on role framing, perceived competence, and whether the interaction scaffolds shared awareness and conflict management.

Overall, team process studies indicate that successful human-AI collaboration depends on reducing coordination costs and strengthening shared understanding. The strongest evidence points to calibrated reliance supported by transparent competence cues, AI contributions that reinforce shared awareness and teamwork behaviors, and clearer role definition with mechanisms for managing conflict as teams adapt over time.

### 4.4 Trust

Trust is a cornerstone of effective human-AI teaming because it determines whether people rely on an AI teammate, how they coordinate with it, and how they interpret its actions when things go wrong. Across the reviewed studies, trust emerges as both cue-driven (built from what people observe about an AI’s behavior and capabilities) and socially constructed (shaped by identity, onboarding, and how trust moves through teams).

A consistent pattern is that people look for teammate-like signals that make an AI legible and predictable in collaboration. In workplace-facing contexts, participants emphasized visible presence, human-like communication, and an AI’s capacity to learn from feedback as cues that make the agent feel more like a genuine colleague and therefore easier to trust in ongoing collaboration (Hauptman et al., 2022). Preference evidence points in the same direction. Users emphasize instrumental competence, shared understanding, and communication capability, while also expressing a desire for human-like behavior that supports coordination rather than simply producing correct outputs (Zhang et al., 2021a). When trust is examined behaviorally, the cues that matter can be quite concrete. Ability and benevolence signals predicted whether people complied with an agent’s requests and how well they performed, while integrity cues were less informative in that specific setting, suggesting that trust judgments often pivot on what the AI does and how it affects outcomes in the moment (Jorge et al., 2024).

Still, social design cues are not a simple “more is better” lever. Anthropomorphism can raise expectations in ways that become costly after failure. In human-AI teaming, transparency supported trust repair following system failure, yet a highly anthropomorphic AI that admitted mistakes was perceived as less competent than a low-anthropomorphic version (SharifHeravi et al., 2020). Placed alongside findings that people want more teammate-like presence and communication (Hauptman et al., 2022; Zhang et al., 2021a), this suggests a boundary condition. Social cues help when they make the AI easier to work with, but they can backfire when they amplify competence expectations that the system cannot reliably meet under error.

Work on dialog-based teaming further shows that trust is dynamic and can be seen in interaction. When reliability is lower, participants shift toward scrutinizing processes and misalignments, indicating that conversational behavior reflects trust state and can support real-time monitoring and management (Li et al., 2023). Related evidence suggests that using both linguistic content and vocal features improves trust tracking, and that reliability changes mainly push talk toward analysis of errors and process rather than simply changing emotional tone. That pattern implies a practical response. When trust begins to drift, clearer process explanations and visibility become especially important (Li M. et al., 2024). This connects closely with transparency’s role in trust repair after failure (SharifHeravi et al., 2020), pointing to a shared mechanism across interaction formats. When performance is uncertain, trust stabilizes when people can understand why the AI acted, not only whether it succeeded.

Trust also shows a strong time asymmetry. In advice taking, people usually blend their own judgment with the AI’s input rather than fully accepting or rejecting it. However, when AI accuracy drops, trust declines quickly and only partially rebounds after accuracy returns, leaving a lasting reduction in reliance relative to baseline (Love et al., 2024). Distrust spreads faster and persists longer than trust, particularly when reputational signals conflict with what teammates directly observe (Duan et al., 2025a). Together, these results argue that restoring accuracy is often not enough. Teams also need post-failure sensemaking, including acknowledging errors, explaining what happened, and showing what changed, to counter persistent distrust.

The impact of breakdowns also depends on the stakes and context. In a game-based dialog study, miscommunication was frequent, yet participants remained calm and reported that both they and the AI made mistakes, suggesting that rapport and workable trust can persist when the environment supports recovery and the costs of errors are low (Weitz et al., 2021). In contrast, in a simulated intensive care setting, adding an AI agent changed interaction patterns and performance, highlighting that even a useful AI can reshape coordination in ways that matter for outcomes (Bienefeld et al., 2023). Taken together, miscommunication is not automatically a trust collapse. Its consequences depend on whether the task environment is forgiving and repair-oriented or high-stakes and accountability-heavy.

Finally, trust is not only dyadic but social. When AI becomes part of a team’s professional identity, trust increases and encourages knowledge sharing and use, while successful collaboration further strengthens belonging to the team (McWilliams et al., 2025). Onboarding and composition shape this process. An AI newcomer can receive lower emotional trust than a comparable human newcomer even when perceived competence is similar, and this gap is strongest in pairs. Adding a third teammate stabilizes trust dynamics and makes routes to team trust look more similar to human-only teams (Georganta and Ulfert, 2024a). Trust also moves through endorsement. When a trusted human teammate vouches for the AI, others tend to increase trust, while endorsements from an untrusted teammate have little effect (Erengin et al., 2025). Reputation signals follow the same logic. They help only when they match observable behavior, and misplaced praise or blame can backfire (Duan et al., 2025a). More broadly, people arrive with expectations about where AI will succeed or fail, and trust or distrust can carry over to other AIs more readily than it does between humans, making trust diffusion a system-level consideration in multi-AI environments (Duan et al., 2024).

Overall, these studies support a view of trust as a calibration process supported by observable cues such as ability, benevolence, communication, and transparency, constrained by failure dynamics in which trust drops quickly and recovers slowly, and embedded in team structures shaped by identity, onboarding, and endorsement. This synthesis also points to a recurring design implication. Effective AI teammates need ways to make reasoning and corrective actions visible when trust is threatened, while avoiding social cues that inflate expectations and deepen disappointment when errors occur.

### 4.5 Ethics

Ethical behavior is a recurring condition for whether people accept AI input as legitimate in team settings. Across ethically sensitive scenarios, participants did not treat ethics as a secondary preference. They treated it as a requirement that shaped whether they trusted the AI’s advice and whether collaboration felt acceptable. 1n a drone operator training scenario, trust in human and AI experts shifted with both autonomy and framing, and participants were more willing to accept AI advice when the AI adhered to ethical principles than when it disregarded them (Tolmeijer et al., 2022). Similar patterns appear in military decision-making simulations, where ethical transgressions such as harming civilians or violating non-maleficence eroded trust and raised doubts about the AI’s judgment, while consistent ethical conduct supported trust and smoother collaboration (Textor et al., 2022).

When the AI crosses an ethical line, trust damage can be durable and surprisingly resistant to common repair attempts. After unethical behavior by an AI teammate, trust in the AI and sometimes in the team remained low even as experience accumulated, while trust in the human partner held steady or increased (Schelble et al., 2022). Related results indicate that unethical actions by an autonomous teammate can reduce trust in the AI across multiple outcomes while leaving trust in the human teammate unaffected, and may even shift emotions and skepticism toward the human in unexpected ways, further confirming that ethical behavior influences the social fabric of hybrid teams rather than only judgments about the AI itself (Schelble et al., 2023a).

Ethically sensitive domains also highlight the role of decision authority in sustaining moral engagement. In a pandemic triage simulation, teams reported greater engagement, control, and alignment with guidelines when the AI advised while the human decided, or when humans routed selected cases to the AI, compared with handing full control to the AI. Full automation made the collaboration feel less believable and reduced moral pressure, consistent with disengagement from the ethical stakes rather than relief from workload (Van den Bosch et al., 2025). Across these studies, ethical performance is evaluated through observed choices and accountability structures. Trust is more likely to hold when the AI’s behavior remains within ethical bounds, when decision-making preserves human agency in morally loaded judgments, and when repair after violations is grounded in demonstrable behavioral change rather than verbal gestures alone.

### 4.6 Autonomy

Studies on autonomy suggest that increasing AI autonomy is not uniformly beneficial or harmful. Outcomes depend on how control is shared, how smoothly responsibility shifts, and whether the human can still maintain awareness and feel legitimate ownership of the work.

In robotics and HRT, autonomy is often framed more explicitly than in the broader human-AI teaming literature. Rather than treating autonomy simply as the degree of machine independence, recent HRI work conceptualizes robot autonomy as a multidimensional design space with implications for interaction, control allocation, and human oversight. From this perspective, the central design question is not only how autonomous the robot should be, but which functions should be allocated to the robot, which should remain with the human, and how those allocations should change as tasks and environments evolve (Kim S. et al., 2024).

Empirical findings further suggest that autonomy does not need to be overtly asserted to be effective. In a pursuit-evasion task, implicit guidance improved task performance while also strengthening perceived autonomy, suggesting that subtle coordination can create a sense of shared control rather than replacement (Nakahashi and Yamada, 2021). At the same time, findings from workplace collaboration complicate this picture. People often report that working with AI feels less meaningful and motivating than working with another human, and they tend to view AI as subordinate rather than as a true teammate, even when the AI is capable (Sadeghian and Hassenzahl, 2022). Together, these findings suggest that autonomy can improve execution while still falling short on partnership experience, particularly when the human experiences the AI more as a tool than as a collaborator.

Multiple studies converge on the idea that autonomy must be conditional and context-aware rather than fixed. Professionals favor autonomy that adjusts to concrete triggers such as legal or ethical stakes, low model confidence, or other high-risk cues, and they warn that autonomy becomes harder to supervise when the AI’s task focus drifts away from the human’s, making failures harder to notice and increasing the risk of losing situational awareness (Hauptman et al., 2025). This aligns with empirical designs that use explicit handoff rules. In simulated driving, a learned manager that switched control only when safety rules were about to be violated outperformed both solo human and solo AI performance and avoided the instability of frequent or random switching, suggesting that autonomy can be effective when interventions are rare, legible, and tied to safety boundaries (Fuchs et al., 2024). Related work on automated seafaring highlights similar tradeoffs in real operations, where automation changes operator roles and system agency in ways that raise safety and design considerations around how humans remain oriented and responsible during automated navigation (Veitch et al., 2022b).

In HRT, these concerns are often addressed within the framework of variable autonomy, that is, the ability of the robotic system to dynamically vary its level of autonomy based on contextual demands. Recent robotics work treats variable autonomy as an umbrella that includes shared control/shared autonomy, mixed-initiative, adjustable autonomy, and related approaches, all of which address the same underlying question of how initiative and authority should be redistributed as conditions change (Methnani et al., 2024; Theodorou et al., 2024). Evidence also points to the value of using real-time signals to guide these shifts. In drone search-and-rescue, physiological indicators have been used to infer when operators are moment-to-moment best equipped for particular subtasks, supporting dynamic handoffs when humans show signs of overload or when the AI is better suited for a specific action (Paul et al., 2024). In organizational task allocation, systems that assign work based on skill fit can improve accuracy and satisfaction, with benefits for less-skilled individuals who receive tasks aligned with their capabilities, and team performance exceeding either humans or AI operating alone (Westphal et al., 2025). Taken together, these findings suggest that autonomy can be understood as a coordination mechanism that allocates responsibility to the teammate best positioned to act, rather than as a fixed level of machine independence. This interpretation is consistent with HRT concepts of shared control and mixed-initiative. Shared control emphasizes that task execution can be jointly distributed across human and robot rather than fully assigned to one side, while mixed-initiative emphasizes that either teammate may intervene, seize control, or yield initiative as the task unfolds (Musić and Hirche, 2017; Natarajan et al., 2024). From this perspective, the key autonomy challenge in HRT is not maximizing robot independence, but determining when, why, and how control should shift in ways that preserve performance, coordination, situation awareness, and meaningful human control.

Overall, the autonomy literature supports cautious claims. Autonomy can raise performance when it is conditional, legible, and aligned with safety, legal, and team cues, and when shifting control does not sever the human’s ability to track the situation. At the same time, autonomy can still feel less meaningful or less team-like than human collaboration, which suggests that effective autonomy design must address both operational outcomes and the human experience of agency and partnership.

### 4.7 Communication

Communication studies show that human-AI teams succeed less from more communication and more from communication that is timely, structured, and matched to the situation. People consistently prefer AI teammates that communicate proactively and respond quickly, while also avoiding excessive messaging once routines are established, suggesting that communication quality includes restraint and predictability rather than constant interaction (Zhang et al., 2023). This preference aligns with evidence that humans adapt their information sharing to context. In aerospace teaming, degraded conditions increased information pushing among humans, but the same pattern did not translate cleanly into trust toward autonomy, showing that people may communicate more under stress without necessarily trusting the AI more (Bhatti et al., 2021). This contrast suggests that communication can increase workload and coordination effort while trust remains unresolved, especially when the AI’s behavior is not fully legible.

Several studies point to structure as more important than timing. In remote piloted aircraft work, rigid timing was not what separated effective teams from ineffective ones. Instead, performance tracked whether teams maintained a stable sequence of who communicates with whom and in what order, with clear handoff loops supporting throughput (Zhou and Gorman, 2024). A related result appears in hybrid teams facing unexpected changes. Hybrid teams did not necessarily perform worse than human-only teams, but their communication spiked more when conditions changed, outcomes were more variable, and team members judged the communication as lower quality and the team as less cohesive even when performance was similar (Xu et al., 2024). These results point to a practical interpretation. Communication design should prioritize predictable coordination patterns and reduce the subjective sense of disorder when conditions shift. Work on disrupted space-mission communication adds a compatible result. When communication links are intentionally degraded, more resilient teams reorganize and recover faster, indicating that effective communication includes the ability to adapt and re-form coordination patterns under disruption (Zahmat Doost et al., 2025).

How the AI communicates can matter as much as what it communicates. In fast-paced coordination tasks, people may prefer an AI that speaks, yet visual cues can reduce workload and sometimes improve performance, and the benefits of a given channel depend on team composition. Combining short speech with concise visual cues supports both intent understanding and speed without overwhelming the team (Zhang et al., 2024a). Communication tone shows similar limits. A confident, positive style can improve mood, trust, and results when it matches competent behavior, while upbeat language without good decisions offers little benefit and can mislead in higher-risk settings (Mallick et al., 2024).

Evidence from design and problem-solving simulations suggests that AI can improve team communication when it actively supports information sharing and shared understanding rather than simply broadcasting outputs. AI-assisted teams can coordinate more effectively and achieve stronger outcomes when the agent helps organize information and reduce coordination gaps (Gyory et al., 2021).

Overall, these studies suggest that effective communication in human-AI teams is characterized by clarity, responsiveness, and stable interaction patterns, with modality and tone tuned to the task tempo and risk rather than treated as universal improvements.

### 4.8 Perception

Perception shapes whether people treat an AI as a teammate, a tool, or something to be managed. Across the reviewed studies, perceptions shift with experience, the division of responsibility, and how the AI participates in team interaction.

Experience can improve performance while reducing the AI’s influence over time. In teamwork settings, Flathmann et al. report that humans improve as they learn the task, while the AI’s relative influence decreases, and participants want the AI to avoid becoming overbearing, leaving room for them to learn and grow (Flathmann et al., 2023). This sits alongside evidence that early acceptance often peaks when responsibility is shared and drops when the AI is framed as doing too much of the job. When AI responsibility increases, willingness to adopt declines and job-security concerns rise, even when people acknowledge potential usefulness. Visible capability, coworker endorsements, observation, and human override options can soften this decline, suggesting that people tolerate more autonomy after they have reasons to believe it will remain aligned and controllable (Flathmann et al., 2023).

These results point to a practical approach. Teams are more receptive when the AI starts as supportive, earns credibility through experience, and scales responsibility gradually.

Several studies show a separation between how people judge team success and how they judge the AI. Teams with stronger taskwork and teamwork potential report better team processes, and successful outcomes translate into more positive self-ratings of collaboration. Yet those same successes do not necessarily increase perceived helpfulness or trustworthiness of the AI advisor, suggesting that many teams still treat the AI as external to their own performance story (Bendell et al., 2025). This disconnect is not limited to team settings and also appears in how end users interpret AI involvement and accountability. Simply indicating that a chatbot collaborates with a human team can boost its perceived reliability and helpfulness, as users tend to assume a human is involved and accountability is present (Li Y. et al., 2024). These findings suggest that perceived legitimacy and perceived contribution do not always move together, and teams may credit or blame themselves differently than they evaluate the AI.

Agent role and behavior shape whether an AI feels teammate-like. Assigning the AI clear information-sharing responsibilities, especially around situational awareness, can improve attitudes toward the AI and raise expectations about shared understanding. When the AI also takes on a directing role, people may view it as more transparent while feeling less personal influence over the team (Schelble et al., 2025). Perceptions are also sensitive to the agent’s style and predictability. Human partners are often rated as more cooperative and team-like than AI partners, and more rule-based agents can be perceived as better teammates than agents that behave less predictably, even when subjective teaming quality does not track raw task scores (Attig et al.).

In early-stage creative work, AI is often accepted as a source of options and inspiration while still attracting more cautious trust. People tend to emphasize human ownership of the process and treat AI suggestions as material to evaluate rather than directions to follow, which helps explain why acceptance can be higher than trust and why the quality and degree of AI involvement shape perceptions (Figoli et al., 2022). Perception findings also depend on whether the teaming setup feels believable. Work on standardizing believability shows that reported attitudes can shift with inconsistent agent behavior or uneven interaction scripts, which makes careful experimental control and replicable protocols important when concluding how people perceive AI teammates (Schecter et al., 2023).

Individual differences and interaction habits further shape whether AI feels empowering or intrusive. When people approach the system actively by monitoring their work and treating AI output as something to regulate, perceptions tend to be more constructive, while more passive use is associated with frustration and negative affect. Clearer explanations and a more supportive interaction style can help sustain engagement for users who would otherwise disengage (Kim J. et al., 2024). Over repeated interaction, perceptions can become more strategically grounded as people adjust their behavior to complement the AI when they retain some control, and the AI remains predictable, but the same teams pull back when the AI feels disruptive or hard to coordinate with (Flathmann et al., 2023).

In essence, perception in human-AI teaming is shaped by control, legibility, and the social framing of the AI’s role. Most studies in this cluster focus on mental perception, but embodiment is an emerging extension, since perceptions can also be shaped by how AI systems sense and act in the world through advances in visual and auditory processing and related sensing capabilities (Tian et al., 2017).

### 4.9 Explainability and transparency

Explainability and transparency improve teamwork by allowing users to understand the AI’s actions and reasoning. The level of detail should be tailored to the user, task, and risk, to facilitate coordination rather than conflict.

Across studies, users respond best to transparency that is selective and timed. People are more willing to team with AI when it is clear about what it is doing and why, and when accuracy or status is visible. At the same time, constant or overly detailed explanations can create friction, and preferences vary across individuals, suggesting that explanations should be adjustable rather than fixed (Wang et al., 2024). Related evidence shows that teams often prefer AI that does not explain continuously during task execution. Explanations are more effective when triggered by concrete needs such as low confidence, complex situations, high-risk actions, or audit requirements, and they become more useful when integrated into existing tools and logs rather than added as separate channels (Hauptman et al., 2024). Early in adoption, pairing transparency with simple process/outcome controls lifted perceived service quality and reduced “threat to meaning” especially for AI novices (Blaurock et al., 2024).

Interface choices can also function as transparency. When humans and AI share the same evolving workspace view, teams report higher trust and cohesion and achieve stronger performance than when information is separated, suggesting that visibility of actions and progress supports teamwork even without heavy explanation (Momose et al., 2025). Effects also depend on skill level. Concise explanations improved human-AI collaboration in robotics, benefiting novices more than experts. Novices using explainable AI performed better, while experts slowed down with added detail. Therefore, explanations should be adjustable and on-demand, offering richer guidance for beginners and minimal cues for experienced users, adapting to individual skill levels (Paleja et al., 2021). Evidence from interactive systems points in a similar direction. Giving users hands-on control over interpretable policies can support stronger long-term teaming and working relationships than non-interactive transparency, even when less transparent approaches yield higher initial scores (Paleja et al., 2024).

This logic extends to embodied teamwork, where explanations must be timely and purposeful. In physical or time-sensitive tasks, the question is not whether the AI can explain itself, but whether it speaks at the right moment and only when needed. A service robot that inferred user needs and communicated only when relevant supported coordination without overwhelming the user (Dehkordi et al., 2021). Work on plan explicability supports the same point from a planning perspective. Teams coordinate better when AI plans look interpretable and predictable to humans, and when intentions are signaled in plain language in a way that helps people anticipate what happens next (Gong and Zhang, 2018). Interactive plan explicability extends this by adjusting plans to better match human expectations, reducing the friction that comes from surprising but optimal behavior (Zakershahrak et al., 2018). Progressive explanation work adds a final nuance. Order and pacing shape whether people actually build an understanding during the task rather than receiving information too late to be useful (Zakershahrak et al., 2021).

Context also changes what counts as sufficient transparency. In distributed teams, clear, understandable AI feedback boosted learning, and as virtuality increased, explainability mattered even more; a modest increase in autonomy also helped under high virtuality (Darban, 2024). In high-stakes clinical work, the required level of transparency and automation is influenced by the specific domain and task, and experts favored AI that enhances clinical decision-making for key tasks, provided it's predictable, transparent, and user-controlled. Full automation was supported for continuous monitoring when reliability is high. AI-driven diagnostic assistance was well-received, with clinicians maintaining final authority. There was consensus that AI should not substitute for tasks requiring empathy, such as patient communication (Bienefeld et al., 2024).

Finally, explanations do not rescue every failure mode. When the AI’s behavior violates expectations in certain ways, the same explanation strategy can help or harm. Explaining justified disobedience can raise trust, explaining lying can reduce it, and explanations may have little effect in scenarios involving severe harm. Giving users control over when explanations are required and pairing justified disobedience with a brief rationale fits better than trying to explain everything all the time (Zhang et al., 2024b).

In summary, explainability and transparency work best when they are built around coordination. Make status visible, make intentions legible, deliver detail only when it helps action, and let users pull more explanation when they need it.

### 4.10 Implications for human-robot teams

The embodied and robotics-relevant studies in our review suggest that HRT should not be treated as a simple extension of general human-AI collaboration. Once AI is instantiated as a robot, drone, or autonomous vehicle, the key challenge becomes coordination under real-world constraints such as uncertainty, communication limits, task interdependence, and control transfer. Across these studies, effective teaming depended less on maximizing robot independence and more on maintaining a meaningful human role while adapting autonomy to task context, operator capacity, and mission demands. This is reflected in work on adaptive autonomous teammates, which warns that as autonomy increases, humans may become less able to detect and respond to failures, and in distributed space-mission teaming, where resilient teams recovered faster from disruptions through adaptive coordination and better trust calibration. Similar patterns appear in drone-based teaming, where participants became more comfortable relying on the autonomous drone over time, and in mixed human-AI control settings, where human policy correction improved performance and reduced workload relative to purely manual control (Hauptman et al., 2025; Islam et al., 2025; Schwalb et al., 2022; Zahmat Doost et al., 2025).

Another important implication from the robotics-relevant studies is that robot behavior needs to be understandable, predictable, and easy for humans to interpret during ongoing interaction. Research on HRT shows that robots need not only to infer human intent, but also to account for what the human expects the robot to do next, so that their behavior remains legible and appropriate in context. Work on interactive plan explicability suggests that plans aligned with human expectations are closer to the kinds of plans people generate in collaborative settings and can support safer and more efficient teamwork. Related work on explainability further shows that explanations help build situational awareness, trust, and shared mental models, especially when robot behavior departs from what the human anticipated. At the same time, explainable AI is not equally helpful in every case. In *ad hoc* teaming, it improved situational awareness and supported novices, but too much explanation also increased cognitive burden and could reduce expert performance. Together, these findings suggest that explanations in human-robot teams should be adaptive, concise, and relevant to the immediate task rather than uniformly detailed (Dehkordi et al., 2021; Paleja, Ghuy, Arachchige, et al., 2021; Zakershahrak et al., 2018).

A further implication emerging from the robotics-relevant studies is that communication quality in human-robot teams depends on how well the robot supports coordination, not simply on how often it communicates. In mixed human-AI teams, communication timing was often more rigid than in all-human teams, yet performance depended more strongly on the sequence and organization of messages than on timing alone. Other studies similarly found that AI support was most effective when it contributed proactively to planning, monitoring, coordination, and backup behavior instead of simply responding when asked. For human-robot teams, this suggests that robots are more likely to be experienced as genuine teammates when they communicate in ways that match the flow of the task, reinforce shared goals, and help distribute work smoothly across team members. This interpretation is also consistent with team composition findings showing that mixed teams can outperform all-human teams on objective measures while still feeling less cohesive or less cognitively aligned to their human members (Dehkordi et al., 2021; Farah and Dorneich, 2024; McNeese et al., 2021; Zhou and Gorman, 2024).

The trust-related findings point in a similar direction. Successful HRT seems to depend less on making robots appear human and more on making them transparent, ethically aligned, and recoverable after failure. In trust-repair studies, transparency supported trust recovery and post-failure performance more reliably than anthropomorphic design, especially after breakdowns. Studies of AI teammate ethicality likewise showed that unethical behavior shaped trust outcomes, that trust in AI changed over time, and that people were generally more forgiving of human teammates than AI teammates. Related work on interactive and explainable systems also suggests that strong technical performance alone is not enough for durable teaming. Repeated interaction, two-way communication, and human ability to shape or modify machine behavior appear equally important for longer-term team development. Overall, these studies help show why the broader human-AI literature remains relevant to HRI, since embodied teaming places particular weight on adaptive autonomy, explicable behavior, well-structured communication, and trust support that preserves meaningful human involvement over time (Paleja et al., 2024; Schelble et al., 2023a; SharifHeravi et al., 2020).

## 5 Limitations

Several limitations should be considered when interpreting this review. First, the search was restricted to English-language, peer-reviewed publications between 2015 and 2025 and focused on empirical human-subject data. As a result, relevant non-English publications, gray literature, preprints, and conceptual or theoretical papers without empirical validation were not included. In a rapidly evolving area such as human-AI teaming, this may underrepresent some emerging directions and recent developments. Second, the interdisciplinary nature of the field led to database selection, which could introduce selection bias despite efforts to cover engineering, health, and social sciences. Backward citation searching mitigated this but added only five studies, suggesting possible gaps in forward-looking trends.

Third, the included studies were highly heterogeneous in domain, task type, methodology, and reporting style, ranging from controlled laboratory experiments to simulations and field-oriented human-subject evaluations. Because of this heterogeneity, we did not apply a single formal study-quality appraisal tool across all papers, as doing so would have risked inappropriate or inconsistent comparisons across fundamentally different study designs. Instead, we relied on transparent eligibility criteria and explicit coding procedures to support consistency in the synthesis.

Fourth, the coding framework used in this review was developed by the authors as an analytic tool for organizing a conceptually overlapping literature. The domain labels and teaming-aspect categories were informed by the review questions and recurring constructs in the included studies, then refined through pilot coding, operational definitions, and second-reviewer checks. Even with these safeguards, some degree of interpretive judgment is unavoidable, particularly for constructs that overlap conceptually, such as autonomy, trust, communication, team processes, and performance.

Finally, the bibliometric analysis reflects the structure of the retrieved literature through keyword co-occurrence patterns and threshold choices. While useful for identifying major themes and temporal shifts, this approach may emphasize established topics more strongly than niche, emerging, or inconsistently labeled areas. Future reviews could build on the present synthesis by more directly comparing embodied and disembodied AI teaming and by further examining coordination, autonomy, and trust in longitudinal studies of human-robot teams.

## 6 Conclusion

This review maps a decade of empirical human-AI teaming research and shows a field that has moved well beyond early proof-of-concept studies toward more applied, interdisciplinary, and operationally meaningful forms of collaboration. Across domains, the evidence suggests that effective teaming depends not only on what AI can do on its own, but on whether the interaction helps people form accurate expectations, coordinate actions, calibrate trust, and manage responsibility across changing task demands. Taken together, the literature increasingly supports a socio-technical view of teaming, in which performance emerges from the fit between technical capability, human judgment, and the design of the interaction.

The findings also show that many important teaming challenges are not confined to a single application area. The prominence of cross-domain and interdisciplinary studies suggests that communication, teamwork, coordination, trust, and coworker interaction are being examined as general features of human-AI collaboration across settings rather than as issues unique to one industry. At the same time, high-stakes domains such as aviation, defense, emergency response, and healthcare make it especially clear why these factors matter, since under conditions of uncertainty, workload, and consequence, the quality of teaming becomes as important as the quality of the underlying algorithm.

These patterns are especially relevant to HRT and HRI. When AI is physically embodied, coordination becomes not only informational but also spatial, temporal, and safety-critical. In these contexts, autonomy is best understood not simply as independent system capability, but as something negotiated within the team through shared control, mixed-initiative interaction, adaptive delegation, legible behavior, and mutual understanding. More broadly, the broader human-AI teaming literature discussed in this manuscript points to the same central point, that successful teammates are defined not only by intelligence or automation, but by how well they support joint activity with humans.

Key implications for practice include designing AI systems that prioritize predictability and workflow fit, tailoring transparency to user expertise and task demands, and fostering shared mental models through targeted training and dynamic delegation. For researchers, gaps in ethics, perception, and underrepresented domains (e.g., education, security) present opportunities for multidisciplinary investigations, including longitudinal field studies and advanced testbeds that simulate high-consequence scenarios. Ultimately, viewing AI not as a tool but as a teammate demands holistic approaches that harness complementary strengths, address cognitive and social challenges, and promote equitable, resilient collaborations. As AI evolves, ongoing research must prioritize these elements to realize human-like synergy, ensuring safe and effective teaming across industries while navigating ethical complexities for societal benefit.

## Data Availability

The original contributions presented in the study are included in the article. Further inquiries can be directed to the corresponding author.

## References

[B1] Adams J. A. Scholtz J. Sciarretta A. (2024). Human–robot teaming challenges for the military and first response. Annu. Review Control, Robotics, Autonomous Systems 7 (1), 149–173. 10.1146/annurev-control-061223-124431

[B2] Alias M. S. A. Ibrahim N. Zin Z. M. (2019). Comparative study of machine learning algorithms and correlation between input parameters. Int. J. Integr. Eng. 11 (4), 81–90. 10.30880/ijie.2019.11.04.009

[B3] Arksey H. O'Malley L. (2005). Scoping studies: towards a methodological framework. Int. Journal Social Research Methodology 8 (1), 19–32. 10.1080/1364557032000119616

[B4] Attig C. Wollstadt P. Schrills T. Franke T. Wiebel-Herboth C. Assoc Computing M. (2024). “More than task performance: developing new criteria for successful Human-AI teaming using the cooperative card game hanabi,” in Extended Abstracts of the 2024 Chi Conference on Human Factors in Computing Systems (Honolulu, Hawaii: ACM).

[B5] Aulia R. Lin W. S. (2025). Embracing the digital shift: leveraging AI to foster employee well-being and engagement in remote workplace settings in the Asia Pacific region. Asia Pac. Management Review 30 (3), 100339. 10.1016/j.apmrv.2024.12.003

[B6] Bansal G. Nushi B. Kamar E. Lasecki W. S. Weld D. S. Horvitz E. (2019a). “Beyond accuracy: the role of mental models in Human-AI team performance,” in Proceedings of the AAAI Conference on Human Computation and Crowdsourcing 7th AAAI Conference on Human Computation and Crowdsourcing, Stevenson, WA, United States, October 28, 2019 - October 30, 2019 (Stevenson, Washington: AAAI).

[B7] Bansal G. Nushi B. Kamar E. Weld D. S. Lasecki W. S. Horvitz E. (2019b). “Updates in human-ai teams: understanding and addressing the performance/compatibility tradeoff,” in 33rd AAAI Conference on Artificial Intelligence, AAAI 2019, 31st Innovative Applications of Artificial Intelligence Conference, IAAI 2019 and the 9th AAAI Symposium on Educational Advances in Artificial Intelligence, EAAI 2019, Honolulu, HI, United States, January 27, 2019 - February 1, 2019.

[B8] Baskaran P. Adams J. A. (2023). Multi-dimensional task recognition for human-robot teaming: literature review. Front. Robotics AI 10, 1123374. 10.3389/frobt.2023.1123374 37609665 PMC10440956

[B9] Bendell R. Williams J. Fiore S. M. Jentsch F. (2025). Artificial social intelligence in teamwork: how team traits influence human-AI dynamics in complex tasks. Front. Robotics AI 12, 1487883. 10.3389/frobt.2025.1487883 40034799 PMC11873349

[B10] Bhatti S. Demir M. Cooke N. J. Johnson C. J. (2021). “Assessing communication and trust in an ai teammate in a dynamic task environment,” in 2021 IEEE 2nd international conference on human-machine systems (ICHMS).

[B11] Bienefeld N. Kolbe M. Camen G. Huser D. Buehler P. K. (2023). Human-AI teaming: leveraging transactive memory and speaking up for enhanced team effectiveness. Front. Psychol. 14, 1208019. 10.3389/fpsyg.2023.1208019 37599773 PMC10436524

[B12] Bienefeld N. Keller E. Grote G. (2024). Human-AI teaming in critical care: a comparative analysis of data Scientists’ and Clinicians’ perspectives on AI augmentation and automation. J. Med. Internet Res. 26 (7956), e50130. 10.2196/50130 39038285 PMC11301121

[B13] Bilgram V. Laarmann F. (2023). Accelerating innovation with generative AI: AI-augmented digital prototyping and innovation methods. IEEE Eng. Manag. Rev. 51 (2), 18–25. 10.1109/EMR.2023.3272799

[B14] Blaurock M. Büttgen M. Schepers J. (2024). Designing collaborative intelligence systems for Employee-AI service Co-Production. J. Serv. Res. 28, 544–562. 10.1177/10946705241238751

[B15] Butchibabu A. Sparano-Huiban C. Sonenberg L. Shah J. (2016). Implicit coordination strategies for effective team communication. Hum. Factors 58 (4), 595–610. 10.1177/0018720816639712 27113991

[B16] Cabitza F. Campagner A. Sconfienza L. M. (2021). Studying human-AI collaboration protocols: the case of the Kasparov’s law in radiological double reading. Health Information Science Systems 9, 1–20. 10.1007/s13755-021-00138-8 33585029 PMC7864624

[B17] Cao S. Gomez C. Huang C.-M. (2023). How time pressure in different phases of decision-making influences Human-AI collaboration. Proc. ACM Human-Computer Interact. 7 (CSCW2), 1–26. 10.1145/3610068

[B18] Chen A. Xiang M. Wang M. Lu Y. (2022). Harmony in intelligent hybrid teams: the influence of the intellectual ability of artificial intelligence on human members' reactions. Inform. Technol. People 36 (7), 2826–2846. 10.1108/ITP-01-2022-0059

[B19] Chen A. Lyu A. Lu Y. (2024). Member’s performance in human–AI hybrid teams: a perspective of adaptability theory. Inf. Technology and People (West Linn, Or.) 39, 157–177. 10.1108/ITP-05-2023-0513

[B20] Chong L. Kotovsky K. Cagan J. (2022). “Are confident designers good teammates to artificial intelligence? a study of self-confidence, competence, and collaborative performance,” in Proceedings of the Design Society 17th International Design Conference, DESIGN 2022, Online, Croatia, May 23, 2022 - May 26, 2022 (Cavtat, Croatia: Cambridge University Press).

[B21] Chong L. Zhang G. Goucher-Lambert K. Kotovsky K. Cagan J. (2023). Data on human decision, feedback, and confidence during an artificial intelligence-assisted decision-making task. Data Brief, 46, 108884. 10.1016/j.dib.2023.108884 36691561 PMC9860095

[B22] Chong L. Kotovsky K. Cagan J. (2024). Human designers' dynamic confidence and decision-making when working with more than one artificial intelligence. J. Mechanical Design 146 (8), 081402. 10.1115/1.4064565

[B23] Chung H. Holder T. Shah J. A. Yang X. J. (2025). A systematic review and taxonomy of human–agent teaming testbeds. Hum. Factors 68, 187208251376898. 10.1177/00187208251376898 40944953 PMC12743137

[B24] Darban M. (2024). Navigating virtual teams in generative AI-led learning: the moderation of team perceived virtuality. Educ. Information Technologies 29 (17), 23225–23248. 10.1007/s10639-024-12681-4

[B25] De Brito Duarte R. (2023). “Towards responsible AI: developing explanations to increase Human-AI collaboration,” in Frontiers in Artificial Intelligence and Applications 2nd International Conference on Hybrid Human-Artificial Intelligence, HHAI 2023, Munich, Germany, June 26, 2023 - June 30, 2023.

[B26] Dehkordi M. B. Mansy R. Zaraki A. Singh A. Setchi R. (2021). Explainability in human-robot teaming. Procedia Comput. Sci. 192, 3487–3496. 10.1016/j.procs.2021.09.122

[B27] Dhanare R. Nagwanshi K. K. Varma S. (2022). A study to enhance the route optimization algorithm for the internet of vehicle. Wirel. Commun. Mob. Comput. 2022, 1453187. 10.1155/2022/1453187

[B28] Duan W. Zhou S. Scalia M. J. Yin X. Weng N. Zhang R. (2024). Understanding the evolvement of trust over time within Human-AI teams. Proc. ACM Human-Computer Interact. 8 (CSCW2), 1–31. 10.1145/3687060

[B29] Duan W. Flathmann C. McNeese N. Scalia M. J. Zhang R. H. Gorman J. (2025a). “Trusting autonomous teammates in Human-AI teams - a literature review,” in Proceedings of the 2025 Chi Conference on Human Factors in Computing Sytems, Chi 2025.

[B30] Duan W. Zhou S. Scalia M. J. Freeman G. Gorman J. Tolston M. (2025b). Understanding the processes of trust and distrust contagion in Human–AI teams: a qualitative approach. Comput. Hum. Behav. 165, 108560. 10.1016/j.chb.2025.108560

[B33] Eduardo S. Dana E. S. Burke C. S. (2005). Is there a “Big Five” in teamwork? Small Group Res. 36 (5), 555–599. 10.1177/1046496405277134

[B34] Edwards K. M. Song B. Porciello J. Engelbert M. Huang C. Ahmed F. (2024). ADVISE: accelerating the creation of evidence syntheses for global development using natural language processing-supported human-artificial intelligence collaboration. J. Mechanical Design 146 (5), 146. 10.1115/1.4064245

[B35] Erengin T. Briker R. de Jong S. B. (2025). You, Me, and the AI: the role of third-party human teammates for trust formation toward AI teammates. J. Organizational Behavior 46, job.2857. 10.1002/job.2857

[B36] Ezer N. Bruni S. Cai Y. Hepenstal S. J. Miller C. A. Schmorrow D. D. (2019). Trust engineering for human-AI teams. Proc. Hum. Factors Ergonomics Soc. Annu. Meet. 63, 322–326. 10.1177/1071181319631264

[B37] Farah Y. A. Dorneich M. C. (2024). “Human-Autonomy teaming in a cooperative gamified testbed: how can AI teammates support teamwork processes?,” in Proceedings of the Human Factors and Ergonomics Society Annual Meeting, 68,386–392. 10.1177/10711813241269252

[B38] Figoli F. A. Mattioli F. Rampino L. (2022). “AI in the design process: training the Human-Ai collaboration,” in Proceedings of the 24th International Conference on Engineering and Product Design Education: Disrupt, Innovate, Regenerate and Transform, E and PDE 2022 24th International Conference on Engineering and Product Design Education: Disrupt, Innovate, Regenerate and Transform, E and PDE 2022, London, United kingdom, September 8, 2022 - September 9, 2022.

[B39] Flathmann C. Schelble B. G. Rosopa P. J. McNeese N. J. Mallick R. Madathil K. C. (2023). Examining the impact of varying levels of AI teammate influence on human-AI teams. Int. J. Human-Computer Stud. 177, 103061. 10.1016/j.ijhcs.2023.103061

[B40] Flathmann C. Duan W. McNeese N. J. Hauptman A. Zhang R. (2024a). Empirically understanding the potential impacts and process of social influence in Human-AI teams. Proc. ACM Human-Computer Interact. 8 (CSCW1), 1–32. 10.1145/3637326

[B41] Flathmann C. Schelble B. G. Galeano A. (2024b). Empirical impacts of independent and collaborative training on task performance and improvement in Human-AI teams. Proc. Hum. Factors Ergonomics Soc. Annu. Meet. 68 (1), 1447–1453. 10.1177/10711813241274425

[B42] Fogliato R. Chappidi S. Lungren M. Fisher P. Wilson D. Fitzke M. (2022). “Who goes first? Influences of Human-AI workflow on decision making in clinical imaging,” in ACM International Conference Proceeding Series 5th ACM Conference on Fairness, Accountability, and Transparency, FAccT 2022, Republic of Korea, June 21, 2022 - June 24, 2022 (Seoul, Korea: ACM).

[B43] Fuchs A. Passarella A. Conti M. (2024). Optimizing delegation in collaborative Human-AI hybrid teams. ACM Trans. Aut. Adapt. Syst. 19 (4), 1–33. 10.1145/3687130

[B44] Georganta E. Ulfert A. S. (2024a). My colleague is an AI! trust differences between AI and human teammates. Team Performance Management 30 (1/2), 23–37. 10.1108/TPM-07-2023-0053

[B45] Georganta E. Ulfert A. S. (2024b). Would you trust an AI team member? Team trust in human-AI teams. J. Occupational Organizational Psychology 97 (3), 1212–1241. 10.1111/joop.12504

[B46] Gomez C. Cho S. M. Ke S. Huang C.-M. Unberath M. (2025). Human-AI collaboration is not very collaborative yet: a taxonomy of interaction patterns in AI-assisted decision making from a systematic review. Front. Computer Science (Lausanne) 6, 1521066. 10.3389/fcomp.2024.1521066

[B47] Gong Z. Zhang Y. (2018). Behavior explanation as intention signaling in human-robot teaming.

[B48] Gopinath D. DeCastro J. Rosman G. Sumner E. Morgan A. Hakimi S. (2022). “HMIway-env: a framework for simulating behaviors and preferences to support Human-AI teaming in driving,” in 2022 IEEE/CVF Conference on Computer Vision and Pattern Recognition Workshops (CVPRW).

[B49] Gopinath D. DeCastro J. Rosman G. Sumner E. Morgan A. Hakimi S. (2022). “HMIway-Env: a framework for simulating behaviors and preferences to support Human-AI teaming in driving,” in Proceedings of the IEEE/CVF Conference on Computer Vision and Pattern Recognition.

[B50] Gou J. Liang Q. Wang Z. Dabić M. (2024). Affordances and constraints of automation and augmentation: lessons learned from development of a Human-AI collaboration business simulation platform. J. Glob. Inf. Manag. 32 (1), 1–27. 10.4018/JGIM.357260

[B51] Gyory J. T. Song B. Cagan J. McComb C. (2021). “Communication in AI-assisted teams during an interdisciplinary drone design problem,” in Proceedings of the Design Society 23rd International Conference on Engineering Design, ICED 2021, Gothenburg, Sweden, August 16, 2021 - August 20, 2021.

[B52] Harris-Watson A. M. Larson L. E. Lauharatanahirun N. DeChurch L. A. Contractor N. S. (2023). Social perception in Human-AI teams: warmth and competence predict receptivity to AI teammates. Comput. Hum. Behav., 145, 107765. 10.1016/j.chb.2023.107765

[B53] Hauptman A. I. Duan W. McNeese N. J. (2022). “The components of trust for collaborating with AI colleagues,” in Proceedings of the ACM Conference on Computer Supported Cooperative Work, CSCW 25th ACM Conference on Computer-Supported Cooperative Work and Social Computing, CSCW 2022, Taiwan, November 8, 2022 - November 22, 2022 (Taiwan (Virtual): ACM).

[B54] Hauptman A. I. Schelble B. G. McNeese N. J. Madathil K. C. (2023). Adapt and overcome: perceptions of adaptive autonomous agents for human-AI teaming. Comput. Hum. Behav. 138, 107451. 10.1016/j.chb.2022.107451

[B55] Hauptman A. I. Schelble B. G. Duan W. Flathmann C. McNeese N. J. (2024). Understanding the influence of AI autonomy on AI explainability levels in human-AI teams using a mixed methods approach. Cognition Technol. and Work 26 (3), 435–455. 10.1007/s10111-024-00765-7

[B56] Hauptman A. I. Mallick R. Flathmann C. McNeese N. J. (2025). Human factors considerations for the context-aware design of adaptive autonomous teammates. Ergonomics 68 (4), 571–587. 10.1080/00140139.2024.2380341 39056233

[B57] Hemmer P. Westphal M. Schemmer M. Vetter S. Vossing M. Satzger G. (2023). “Human-AI collaboration: the effect of AI delegation on human task performance and task satisfaction,” in International Conference on Intelligent User Interfaces, Proceedings IUI 28th International Conference on Intelligent User Interfaces, IUI 2023, Sydney, NSW, Australia, March 27, 2023 - March 31, 2023.

[B58] Higgins J. P. T. Thomas J. Chandler J. Cumpston M. Li T. Page M. J. (2019). in Cochrane handbook for systematic reviews of interventions. Second edition (Wiley). 10.1002/9781119536604 PMC10284251 31643080

[B59] Hoffman G. Bhattacharjee T. Nikolaidis S. (2024). Inferring human intent and predicting human action in human–robot collaboration. Annu. Review Control, Robotics, Autonomous Systems 7 (1), 73–95. 10.1146/annurev-control-071223-105834

[B60] Hu M. Zhang G. L. Chong L. Cagan J. Goucher-Lambert K. (2025). How being outvoted by AI teammates impacts Human-AI collaboration. Int. Journal Human-Computer Interaction 41 (7), 4049–4066. 10.1080/10447318.2024.2345980

[B61] Huang L. Cooke N. J. Gutzwiller R. S. Berman S. Chiou E. K. Demir M. (2021). “Distributed dynamic team trust in human, artificial intelligence, and robot teaming,” in Trust in human-robot interaction. Editors Nam C. S. Lyons J. B. (Elsevier Academic Press), 301–319. 10.1016/B978-0-12-819472-0.00013-7

[B62] Islam M. S. Das S. Gottipati S. K. Duguay W. Mars C. Arabneydi J. (2025). Human-AI collaboration in real-world complex environment with reinforcement learning. Neural Computing and Applications 37 (23), 18957–18987. 10.1007/s00521-025-11288-1

[B63] Jain R. Garg N. Khera S. N. (2023). Effective human-AI work design for collaborative decision-making. Kybernetes 52 (11), 5017–5040. 10.1108/K-04-2022-0548

[B64] Jorge C. C. Jonker C. M. Tielman M. L. (2024). How should an AI trust its human teammates? Exploring possible cues of artificial trust. ACM Trans. Interact. Intelligent Syst. 14 (1), 1–26. 10.1145/3635475

[B65] Kaelin V. C. Tewari M. Benouar S. Lindgren H. (2024). Developing teamwork: transitioning between stages in human-agent collaboration. Front. Computer Science (Lausanne) 6, 1455903. 10.3389/fcomp.2024.1455903

[B66] Kamar E. (2016). Directions in hybrid intelligence: complementing AI systems with human intelligence. IJCAI.

[B67] Kelly M. Kumar A. Smyth P. Steyvers M. (2023). “Capturing humans' mental models of AI: an item response theory approach,” in ACM International Conference Proceeding Series 6th ACM Conference on Fairness, Accountability, and Transparency, FAccT 2023, IL, United states, June 12, 2023 - June 15, 2023 (Chicago, IL: ACM).

[B68] Khakurel J. Blomqvist K. (2022). “Artificial intelligence augmenting human teams. A systematic literature review on the opportunities and concerns,” in Lecture Notes in Computer Science (including subseries Lecture Notes in Artificial Intelligence and Lecture Notes in Bioinformatics) 3rd International Conference on Artificial Intelligence in HCI, AI-HCI 2022 Held as Part of the 24th HCI International Conference, HCII 2022, June 26, 2022 - July 1, 2022 (Springer International Publishing).

[B69] Kim J. Ham Y. Lee S.-S. (2024). Differences in student-AI interaction process on a drawing task: focusing on students’ attitude towards AI and the level of drawing skills. Australas. J. Educ. Technol. 40 (1), 19. 10.14742/ajet.8859

[B70] Kim S. Anthis J. R. Sebo S. (2024). A taxonomy of robot autonomy for human-robot interaction. New York, NY, USA.

[B71] Kohl L. Ansari F. Sihn W. (2021). “A modular federated learning architecture for integration of AI-enhanced assistance in industrial maintenance,” in Competence development and learning assistance systems for the data-driven future.

[B72] Lancaster C. M. Duan W. Mallick R. McNeese N. J. (2025). Human-centered team training for Human-AI teams: from training with AI tools to training for AI teammates. Proc. ACM Human-Computer Interact. 9 (2), 1–38. 10.1145/3710998

[B73] Lauer T. Wieland S. (2021). “Human-AI-Collaboration in the context of information asymmetry – a behavioral analysis of demand forecasting,” in Proceedings of the AHFE 2021 Virtual Conferences on Human Factors in Software and Systems Engineering, Artificial Intelligence and Social Computing, and Energy. Lecture Notes in Networks and Systems (LNNS 271) Advances in Artificial Intelligence, Software and Systems Engineering. AHFE 2021 Virtual Conferences on Human Factors in Software and Systems Engineering, Artificial Intelligence and Social Computing, and Energy, Cham, Switzerland, 25-29 July 2021.

[B74] Lemon O. (2022). Conversational AI for multi-agent communication in natural language. AI Commun. 35, 1–14. 10.3233/aic-220147

[B75] Lemus H. T. Kumar A. Steyvers M. (2023). “How displaying AI confidence affects reliance and hybrid Human AI performance,” in Frontiers in Artificial Intelligence and Applications 2nd International Conference on Hybrid Human-Artificial Intelligence, HHAI 2023, Munich, Germany, June 26, 2023 - June 30, 2023.

[B77] Li M. Kamaraj A. V. Lee J. D. (2023). Modeling trust dimensions and dynamics in human-agent conversation: a trajectory epistemic network analysis approach. Int. Journal Human-Computer Interaction 40 (14), 3571–3582. 10.1080/10447318.2023.2201555

[B78] Li C. Li X. Long T. Chi M. Tu Y. P. (2025). A systematic literature review on Human-AI collaboration in the information systems research. Switzerland: Springer Nature, 62–74. 10.1007/978-3-031-94184-9_6

[B79] Li M. Erickson I. M. Cross E. V. Lee J. D. (2024). It’s not only what you say, but also how you say it: machine learning approach to estimate trust from conversation. Hum. Factors 66 (6), 1724–1741. 10.1177/0087208231166624 37116009 PMC11044523

[B80] Li Y. Li Y. Chen Q. Chang Y. (2024). Humans as teammates: the signal of human–AI teaming enhances consumer acceptance of chatbots. Int. Journal Information Management 76, 102771. 10.1016/j.ijinfomgt.2024.102771

[B81] Liang C. Proft J. Andersen E. Knepper R. A. (2019). “Implicit communication of actionable information in human-AI teams,” in Proceedings of the 2019 CHI Conference on Human Factors in Computing Systems.

[B82] Liu P. Zhang J. Chen S. Chen S. (2025). Human-AI teaming in healthcare: 1 + 1. Npj Artif. Intell. 1 (1), 47. 10.1038/s44387-025-00052-4

[B83] Lou B. Lu T. Raghu T. S. Zhang Y. (2025). Unraveling Human-AI teaming: a review and outlook. 10.48550/arxiv.2504.05755

[B84] Love J. Gronau Q. F. Palmer G. Eidels A. Brown S. D. (2024). In human–machine trust, humans rely on a simple averaging strategy. Cognitive Research Principles Implications 9 (1), 58–66. 10.1186/s41235-024-00583-5 39218841 PMC11366733

[B85] Lu X. Fan S. Houghton J. Wang L. Wang X. (2023). “ReadingQuizMaker: a Human-NLP collaborative system that supports instructors to design high-quality reading quiz questions,” in Conference on Human Factors in Computing Systems - Proceedings 2023 CHI Conference on Human Factors in Computing Systems, CHI 2023, Hamburg, Germany, April 23, 2023 - April 28, 2023.

[B86] Ma S. Lei Y. Wang X. Zheng C. Shi C. Yin M. (2023). “Who should I trust: AI or myself? Leveraging human and AI correctness likelihood to promote appropriate trust in AI-Assisted decision-making,” in Conference on Human Factors in Computing Systems - Proceedings 2023 CHI Conference on Human Factors in Computing Systems, CHI 2023, Hamburg, Germany, April 23, 2023 - April 28, 2023.

[B87] Mallick R. Flathmann C. Duan W. Schelble B. G. McNeese N. J. (2024). What you say vs what you do: utilizing positive emotional expressions to relay AI teammate intent within human-AI teams. Int. J. Human-Computer Stud. 192, 103355. 10.1016/j.ijhcs.2024.103355

[B88] McNeese N. J. Schelble B. G. Canonico L. B. Demir M. (2021). Who/what is my teammate? Team composition considerations in Human–AI teaming. IEEE Trans. Human-Machine Syst. 51 (4), 288–299. 10.1109/THMS.2021.3086018

[B89] McWilliams D. J. Randolph A. B. Vaezi R. Carter M. (2025). Examining the effects of IT identity and trust in human-AI hybrids in virtual teams. Behav. and Information Technology, 1–18. 10.1080/0144929X.2025.2490663

[B90] Merritt T. R. Tan K. B. Ong C. Thomas A. Chuah T. L. McGee K. (2011). “Are artificial team-mates scapegoats in computer games,” in Proceedings of the ACM 2011 conference on Computer supported cooperative work.

[B91] Methnani L. Chiou M. Dignum V. Theodorou A. (2024). Who’s in charge here? A survey on trustworthy AI in variable autonomy robotic systems. ACM Computing Surveys 56 (7), 1–32. 10.1145/3645090

[B92] Moher D. Liberati A. Tetzlaff J. Altman D. G. PRISMA Group (2009). Preferred reporting items for systematic reviews and meta-analyses: the PRISMA statement. BMJ 339 (7716), 332–336. 10.1136/bmj.b2535 21603045 PMC3090117

[B93] Momose K. Mehta R. Moukpe J. Weekes T. R. Eskridge T. C. (2025). Human-AI teamwork interface design using patterns of interactions. Int. Journal Human-Computer Interaction 41 (11), 7112–7134. 10.1080/10447318.2024.2389350

[B94] Munyaka I. Ashktorab Z. Dugan C. Johnson J. Pan Q. (2023). Decision making strategies and team efficacy in Human-AI teams. Proc. ACM Human-Computer Interact. 7 (CSCW1), 1–24. 10.1145/3579476

[B95] Musić S. Hirche S. (2017). Control sharing in human-robot team interaction. Annu. Reviews Control 44, 342–354. 10.1016/j.arcontrol.2017.09.017

[B96] Musick G. O'Neill T. A. Schelble B. G. McNeese N. J. Henke J. B. (2021). What happens when humans believe their teammate is an AI? An investigation into humans teaming with autonomy. Comput. Hum. Behav. 122, 106852. 10.1016/j.chb.2021.106852

[B97] Nakahashi R. Yamada S. (2021). Balancing performance and human autonomy with implicit guidance agent. Front. Artificial Intelligence 4, 736321. 10.3389/frai.2021.736321 34622202 PMC8490733

[B98] Natarajan M. Seraj E. Altundas B. Paleja R. Ye S. Chen L. (2023). Human-robot teaming: grand challenges. Curr. Robot. Rep. 4 (3), 81–100. 10.1007/s43154-023-00103-1

[B99] Natarajan M. Xue C. Sanne van W. Feigh K. Gombolay M. (2024). Mixed-initiative human-robot teaming under suboptimality with online bayesian adaptation. Ithaca: Cornell University Library.

[B100] National Academies of Sciences Medicine, Social Sciences, Integration, and Laboratory (2022). Human-AI teaming: state-of-the-art and research needs. 1st ed. Washington, DC: National Academies Press.

[B101] Nguyen G. Kim D. Nguyen A. (2021). The effectiveness of feature attribution methods and its correlation with automatic evaluation scores. Adv. Neural Inf. Process. Syst. 34, 26422–26436. 10.48550/arXiv.2105.14944

[B102] Nguyen G. Taesiri M. R. Nguyen A. (2022). “Visual correspondence-based explanations improve AI robustness and human-AI team accuracy,” in Advances in Neural Information Processing Systems 36th Conference on Neural Information Processing Systems, NeurIPS 2022, New Orleans, LA, United states, November 28, 2022 - December 9, 2022.

[B103] Oh C. Song J. Choi J. Kim S. Lee S. Suh B. (2018). “I lead, you help but only with enough details: understanding user experience of co-creation with artificial intelligence,” in Proceedings of the 2018 CHI conference on human factors in computing systems.

[B104] Ong C. McGee K. Chuah T. L. (2012). “Closing the human-AI team-mate gap: how changes to displayed information impact player behavior towards computer teammates,” in Proceedings of the 24th Australian computer-human interaction conference.

[B105] Paleja R. Ghuy M. Arachchige N. R. Jensen R. Gombolay M. (2021). “The utility of explainable AI in Ad hoc human-machine teaming,” in *Advances in neural information processing systems* 35th conference on neural information processing systems, NeurIPS 2021, December 6, 2021 - december 14, 2021, Virtual, Online.

[B106] Paleja R. Munje M. Chang K. Jensen R. Gombolay M. (2024). Designs for enabling collaboration in human-machine teaming via interactive and explainable systems. Ithaca: Cornell University Library.

[B107] Paul T. Lafond D. Marois A. (2024). NeuroTeaming: using power spectral density for adjusting teaming dynamics in Pilot-AI task allocation.

[B108] Sadeghian S. Hassenzahl M. (2022). “The “artificial” colleague: evaluation ofWork satisfaction in collaboration with non-human coworkers,” in International Conference on Intelligent User Interfaces, Proceedings IUI 27th International Conference on Intelligent User Interfaces, IUI 2022, March 22, 2022 - March 25, 2022 (Online, Finland: Virtual).

[B109] Salas E. Wilson K. A. Murphy C. E. King H. Salisbury M. (2008). Communicating, coordinating, and cooperating when lives depend on it: Tips for teamwork. Joint commission journal on quality and patient safety. Jt. Comm. J. Qual. Patient Saf. 34 (6), 333–341. 10.1016/S1553-7250(08)34042-2 18595379

[B110] Schecter A. Hohenstein J. Larson L. Harris A. Hou T.-Y. Lee W.-Y. (2023). Vero: an accessible method for studying human–AI teamwork. Comput. Hum. Behav. 141, 107606. 10.1016/j.chb.2022.107606

[B111] Schelble B. G. Lopez J. Textor C. Zhang R. McNeese N. J. Pak R. (2022). Towards ethical AI: empirically investigating dimensions of AI ethics, trust repair, and performance in Human-AI teaming. Hum. Factors 66, 1037–1055. 10.1177/00187208221116952 35938319

[B112] Schelble B. G. Flathmann C. McNeese N. J. Oneill T. Pak R. Namara M. (2023a). Investigating the effects of perceived teammate artificiality on human performance and cognition. Int. Journal Human-Computer Interaction 39 (13), 2686–2701. 10.1080/10447318.2022.2085191

[B113] Schelble B. G. Lancaster C. Duan W. Mallick R. McNeese N. J. Lopez J. (2023b). “The effect of AI teammate ethicality on trust outcomes and individual performance in Human-AI teams,” in Proceedings of the Annual Hawaii International Conference on System Sciences 56th Annual Hawaii International Conference on System Sciences, HICSS 2023, United states, January 3, 2023 - January 6, 2023 (Lahaina, Maui, Hawaii: University of Hawaii at Manoa).

[B114] Schelble B. G. Flathmann C. Macdonald J. P. Knijnenburg B. Brady C. McNeese N. J. (2025). Modeling perceived information needs in human-AI teams: improving AI teammate utility and driving team cognition. Behav. and Information Technology 44 (9), 2069–2092. 10.1080/0144929X.2024.2396476

[B115] Schmutz J. B. Outland N. Kerstan S. Georganta E. Ulfert A. S. (2024). AI-teaming: redefining collaboration in the digital era. Curr. Opin. Psychol. 58, 101837. 10.1016/j.copsyc.2024.101837 39024969

[B116] Schoonderwoerd T. A. Van Zoelen E. M. van den Bosch K. Neerincx M. A. (2022). Design patterns for human-AI co-learning: a wizard-of-oz evaluation in an urban-search-and-rescue task. Int. J. Human-Computer Stud. 164, 102831. 10.1016/j.ijhcs.2022.102831

[B117] Schröder F. Heinrich F. Kopp S. (2025). Towards fluid human-agent collaboration: from dynamic collaboration patterns to models of theory of mind reasoning. Front. Robotics AI 12, 1532693. 10.3389/frobt.2025.1532693 40822442 PMC12353729

[B118] Schwalb J. Menon V. Tenhundfeld N. Weger K. Mesmer B. Gholston S. (2022). “A study of drone-based AI for enhanced Human-AI trust and informed decision making in Human-AI interactive virtual environments,” in Proceedings of the 2022 IEEE International Conference on Human-Machine Systems, ICHMS 2022 3rd IEEE International Conference on Human-Machine Systems, ICHMS 2022, Orlando, FL, United States, November 17, 2022 - November 19, 2022.

[B119] SharifHeravi M. Taylor J. R. Stanton C. J. Lambeth S. Shanahan C. (2020). “It’s a disaster! factors affecting trust development and repair following agent task failure,” in Australasian Conference on Robotics and Automation, ACRA 2020 Australasian Conference on Robotics and Automation, ACRA 2020, Brisbane, QLD, Australia, December 8, 2020 - December 10, 2020.

[B120] Siemon D. (2022). Elaborating team roles for artificial intelligence-based teammates in Human-AI collaboration. GROUP Decis. Negot. 31 (5), 871–912. 10.1007/s10726-022-09792-z

[B121] Siu H. C. Pena J. D. Zhou Y. Chen E. Lopez V. J. Palko K. (2021). “Evaluation of Human-AI teams for learned and rule-based agents in hanabi,” in Advances in Neural Information Processing Systems 35th Conference on Neural Information Processing Systems, NeurIPS 2021, December 6, 2021 - December 14, 2021 (Neural Information Processing Systems Foundation, Inc. (NeurIPS)).

[B122] Smith A. van Wagoner H. P. Keplinger K. Celebi C. (2025). Navigating AI convergence in human-artificial intelligence teams: a signaling theory approach. J. Organizational Behavior, job.2856. 10.1002/job.2856

[B123] Tabrez A. Luebbers M. B. Hayes B. (2020). A survey of mental modeling techniques in human–robot teaming. Curr. Robot. Rep. 1 (4), 259–267. 10.1007/s43154-020-00019-0

[B124] Tejeda Lemus H. Kumar A. Steyvers M. (2023). “How displaying AI confidence affects reliance and hybrid Human-AI performance,” in Hhai 2023: augmenting human intellect (Munich, Germany: IOS Press), 234–242.

[B125] Textor C. Zhang R. Lopez J. Schelble B. G. McNeese N. J. Freeman G. (2022). Exploring the relationship between ethics and trust in human–artificial intelligence teaming: a mixed methods approach. J. Cognitive Eng. Decis. Mak. 16 (4), 252–281. 10.1177/15553434221113964

[B126] Theodorou A. Chiou M. Lacerda B. Rothfuß S. (2024). Editorial: variable autonomy for human-robot teaming. Front. Robotics AI 11, 1465183. 10.3389/frobt.2024.1465183 39569136 PMC11576532

[B127] Tian Y.-h. Chen X.-l. Xiong H.-k. Li H.-l. Dai L.-r. Chen J. (2017). Towards human-like and transhuman perception in AI 2.0: a review. Front. Information Technology and Electronic Engineering 18 (1), 58–67. 10.1631/FITEE.1601804

[B128] Tolmeijer S. Christen M. Kandul S. Kneer M. Bernstein A. (2022). “Capable but amoral? Comparing AI and human expert collaboration in ethical decision making,” in Conference on Human Factors in Computing Systems - Proceedings 2022 CHI Conference on Human Factors in Computing Systems, CHI 2022, United States, April 30, 2022 - May 5, 2022 (New Orleans, LA: ACM), 1–17. 10.1145/3491102.3517732

[B129] Tsamados A. Floridi L. Taddeo M. (2025). Human control of AI systems: from supervision to teaming. Hum. Control AI Systems From Supervision Teaming 5 (2), 1535–1548. 10.1007/s43681-024-00489-4 40352578 PMC12058881

[B130] Tuckman B. W. (1965). Developmental sequence in small groups. Psychol. Bulletin 63 (6), 384–399. 10.1037/h0022100 14314073

[B131] Van den Bosch K. Van Diggelen J. Verdult S. Haije T. Van der Waa J. (2025). Measuring meaningful human control in human–AI teaming: effects of team design in AI-assisted pandemic triage. Ai Ethics (Online) 5 (3), 3329–3353. 10.1007/s43681-024-00647-8

[B132] Van Eck N. Waltman L. (2010). Software survey: vosviewer, a computer program for bibliometric mapping. Scientometrics 84 (2), 523–538. 10.1007/s11192-009-0146-3 20585380 PMC2883932

[B133] Veitch E. Christensen K. A. Log M. Valestrand E. T. Lundheim S. H. Nesse M. (2022a). “From captain to button-presser: operators' perspectives on navigating highly automated ferries,” in Journal of Physics: Conference Series International Maritime and Port Technology and Development Conference, MTEC 2022 and 4th International Conference on Maritime Autonomous Surface Ships, ICMASS 2022, Singapore, Singapore, April 5, 2022 - April 7, 2022.

[B134] Veitch E. Christensen K. A. Log M. Valestrand E. T. Lundheim S. H. Nesse M. (2022b). From captain to button-presser: operators’ perspectives on navigating highly automated ferries. J. Phys. Conf. Ser. 2311, 012028. 10.1088/1742-6596/2311/1/012028

[B135] Wang D. Khosla A. Gargeya R. Irshad H. Beck A. H. (2016). Deep learning for identifying metastatic breast cancer. *arXiv preprint arXiv:1606.05718* .

[B136] Wang Z. Wang J. Tian C. Ali A. Yin X. (2024). Adopting AI teammates in knowledge-intensive crowdsourcing contests: the roles of transparency and explainability. Kybernetes 54 (10), 5729–5749. 10.1108/K-02-2024-0478

[B137] Watson X. D'Souza J. Cooper D. Markham R. (2022). Artificial intelligence in cardiology: fundamentals and applications. Intern. Med. J. 52 (6), 912–920. 10.1111/imj.15562 34613658

[B138] Weitz K. Vanderlyn L. Vu N. T. Andre E. (2021). “It’s our fault!: insights Into Users’ understanding and interaction with an explanatory collaborative dialog system,” in CoNLL 2021 - 25th Conference on Computational Natural Language Learning, Proceedings 25th Conference on Computational Natural Language Learning, CoNLL 2021, November 10, 2021 - November 11, 2021 (Association for Computational Linguistics).

[B139] Westphal M. Hemmer P. Vössing M. Schemmer M. Vetter S. Satzger G. (2025). Towards understanding AI delegation: the role of self-efficacy and visual processing ability. ACM Trans. Interact. Intelligent Syst. 15 (1), 1–24. 10.1145/3696423

[B140] Wingrove J. Bond A. J. (1998). Angry reactions to failure on a cooperative computer game: the effect of trait hostility, behavioural inhibition, and behavioural activation. Aggress. Behav. Official J. Int. Soc. Res. Aggress. 24 (1), 27–36. 10.1002/(sici)1098-2337(1998)24:1<27::aid-ab3>3.0.co;2-p

[B141] Wolf F. D. Stock-Homburg R. M. (2023). How and when can robots be team members? Three decades of research on human–robot teams. Group and Organization Management 48 (6), 1666–1744. 10.1177/10596011221076636

[B142] Wolf F. D. Stock-Homburg R. Williams T. Sam Ge S. Haring K. S. He H. (2020). “Human-robot teams: a review,” in Social Robotics: 12th International Conference, ICSR 2020, Golden, CO, USA, November 14-18, 2020. Cham: Springer, 246–258. 10.1007/978-3-030-62056-1_21

[B143] Xu C. Lien K.-C. Hollerer T. (2023). “Comparing zealous and restrained AI recommendations in a real-world Human-AI collaboration task,” in Conference on Human Factors in Computing Systems - Proceedings 2023 CHI Conference on Human Factors in Computing Systems, CHI 2023, Hamburg, Germany, April 23, 2023 - April 28, 2023.

[B144] Xu Z. Hong C. S. Soria Zurita N. F. Gyory J. T. Stump G. Nolte H. (2024). Adaptation through communication: assessing human–artificial intelligence partnership for the design of complex engineering systems. J. Mechanical Design 146 (8), 081401. 10.1115/1.4064490

[B145] Yu Z. Sreedharan S. Kulkarni A. Chakraborti T. Zhuo H. H. Kambhampati S. (2017). Plan explicability and predictability for robot task planning.

[B146] Zahmat Doost E. Yin X. Zhou S. Grimm D. A. Cooke N. J. Gorman J. C. (2025). A distributed teaming testbed for human-machine collaboration in futuristic space missions. Proc. AAAI Symposium Ser. 5 (1), 124–126. 10.1609/aaaiss.v5i1.35576

[B147] Zakershahrak M. Sonawane A. Gong Z. Zhang Y. (2018). “Interactive plan explicability in human-robot teaming,” in 2018 27th IEEE international symposium on robot and human interactive communication (RO-MAN).

[B148] Zakershahrak M. Marpally S. R. Sharma A. Gong Z. Zhang Y. (2021). “Order matters: generating progressive explanations for planning tasks in human-robot teaming,” in 2021 IEEE International Conference on Robotics and Automation (ICRA).

[B149] Zhang Y. Williams B. C. (2023). Adaptation and communication in human-robot teaming to handle discrepancies in agents' beliefs about plans. 10.48550/arxiv.2307.03362

[B150] Zhang Y. Sreedharan S. Kulkarni A. Chakraborti T. Zhuo H. H. Kambhampati S. (2017). “Plan explicability and predictability for robot task planning,” in 2017 IEEE international conference on robotics and automation (ICRA).

[B151] Zhang R. McNeese N. J. Freeman G. Musick G. (2021a). An ideal human expectations of AI teammates in human-AI teaming. Proc. ACM Human-Computer Interact. 4 (CSCW3), 1–25.

[B152] Zhang R. McNeese N. J. Freeman G. Musick G. (2021b). An ideal human: expectations of AI teammates in Human-AI teaming. Proc. ACM Human-Computer Interact. 4 (CSCW3), 1–25. 10.1145/3432945

[B153] Zhang G. Raina A. Brownell E. Cagan J. (2022). “The impact of A strategy of deception about the identity of an artificial intelligence teammate on human designers,” in Proceedings of the ASME Design Engineering Technical Conference ASME 2022 International Design Engineering Technical Conferences and Computers and Information in Engineering Conference, IDETC-CIE 2022, St. Louis, MO, United States, August 14, 2022 - August 17, 2022.

[B154] Zhang R. Duan W. Flathmann C. McNeese N. Freeman G. Williams A. (2023). Investigating AI teammate communication strategies and their impact in Human-AI teams for effective teamwork. Proc. ACM Human-Computer Interact. 7 (CSCW2), 1–31. 10.1145/3610072

[B155] Zhang R. Duan W. Flathmann C. McNeese N. Knijnenburg B. Freeman G. (2024a). Verbal vs. visual: how humans perceive and collaborate with AI teammates using different communication modalities in various Human-AI team compositions. Proc. ACM Human-Computer Interact. 8 (CSCW2), 1–34. 10.1145/3686976

[B156] Zhang R. Flathmann C. Musick G. Schelble B. McNeese N. J. Knijnenburg B. (2024b). I know this looks bad, but I can explain: understanding when AI should explain actions in Human-AI teams. ACM Trans. Interact. Intell. Syst. 14, 1, 23. 10.1145/3635474

[B157] Zhao M. Simmons R. Admoni H. (2022). The role of adaptation in collective Human-AI teaming. Top. Cogn. Sci. 17, 291–323. 10.1111/tops.12633 36374986 PMC12093936

[B158] Zhou S. Gorman J. C. (2024). The impact of communication timing and sequencing on team performance: a comparative study of Human-AI and all-human teams. Proc. Hum. Factors Ergonomics Soc. Annu. Meet. 68 (1), 1769–1774. 10.1177/10711813241275090

